# Evaluating textile waste management policies: Lifecycle gaps and opportunities for improvement

**DOI:** 10.1177/0734242X251393613

**Published:** 2025-11-21

**Authors:** Arun Chandra Manivannan, Anubhuti Bhatnagar, Kirsi Niinimäki, Logeshwaran Panneerselvan, Thava Palanisami

**Affiliations:** 1 Environmental Plastic & Innovation Cluster (EPIC), Global Innovative Centre for Advanced Nanomaterials (GICAN), College of Engineering, Science and Environment, The University of Newcastle, NSW, Australia; 2 Department of Design, Aalto University, Aalto, Finland

**Keywords:** Post-consumer textile waste, fast fashion, extended producer responsibility, textile-specific regulatory framework, textile lifecycle assessment

## Abstract

This review critically examines global governmental initiatives aimed at advancing circular textile waste management, analysing regulatory frameworks, voluntary programs, economic incentives and awareness campaigns. It analyses their scope, intensity and the specific stages of the product lifecycle they target. Europe leads with comprehensive policies such as Extended Producer Responsibility and the Ecodesign for Sustainable Products Regulation, fostering product recyclability and transparency across the textile lifecycle. However, country-specific compliance requirements often pose costly and complex barriers. Oceania’s voluntary initiatives, particularly in Australia, remain limited in scope, while North America is moving toward a structured approach. In contrast, Asian policies prioritize import restrictions over comprehensive waste management. African nations have begun restricting textile waste imports, but face infrastructural gaps in sustainable recycling and production. South America lacks both waste import regulations and initiatives to enhance recycling. This review identifies regulations with a mandatory scope and high-intensity targets as the most effective drivers of circularity but highlights challenges such as regulatory inconsistencies and enforcement difficulties. Implementing waste management regulations that span the entire textile lifecycle, coupled with infrastructure development, incentives for sustainable practices, eco-modulation and domestic recycling support, is key to sustainable textile waste management.

## Introduction

The textile life cycle begins with sourcing raw materials, which vary significantly in their origin and processing. Natural fibres such as cotton, flax or wool are cultivated, harvested and mechanically or chemically processed into spinnable fibres. Synthetic fibres, like polyester or nylon, are chemically synthesized from petrochemical derivatives and extruded into continuous filaments or staple fibres. Subsequent stages of the life cycle include spinning, fabric formation (weaving, knitting, or nonwoven processing), dyeing, finishing and garment manufacturing, followed by distribution, retail, consumer use and maintenance and end-of-life management (reuse, recycling or disposal). Each stage contributes to the industry’s environmental footprint through resource extraction, water and energy consumption, chemical usage, waste generation and greenhouse gas emissions.

This review aims to evaluate and compare governmental policy responses to textile waste across global regions, focusing on how these interventions address key lifecycle stages. By mapping diverse strategies like regulatory and voluntary frameworks to financial incentives, this study provides an insight into how different policy instruments are adopted globally to manage textile waste.

### Environmental impact of textiles across the lifecycle

Cotton production accounts for 3% of irrigation water and 6% of pesticides available globally (Niinimäki et al., 2020). Synthetic fibres derived from petrochemicals have a significant carbon footprint primarily due to the high energy demand during fibre production. One study estimated that producing polyamide fibres consumes approximately 0.51–0.53 gigajoules (Xu et al., 2024) of energy per kilogram, while polyester fibre production requires about 0.32–0.36 GJ/kg (Sandin et al., 2019). These values can vary depending on the source of raw materials, production technology and processing conditions.

The environmental burden intensifies during textile production, particularly in energy and water-intensive stages such as spinning, weaving, dyeing, knitting and nonwoven processing. Among these wet processing steps, especially dyeing and finishing, are major contributors to water use, chemical discharge and energy consumption. The manufacturing phase alone accounts for 20% of global industrial wastewater production (Zhu et al., 2022) and contributes to the depletion of 7% of local groundwater and drinking water resources. Furthermore, substantial quantities of chemicals are employed in textile processing including sizing agents, coatings and dyestuffs (Schönberger, 2019). The global textile supply chain is highly fragmented, with South and Southeast Asian countries serving as the primary producers and developed nations as the largest consumers. This fragmentation underscores the critical role of transportation and logistics in contributing to emissions, though the exact impact remains difficult to quantify due to supply chain complexities.

The use phase of textiles alone contributes around 14% of the total CO_2eq_ emissions in a textile product’s life cycle (Abrishami et al., 2024; Moazzem et al., 2018). Approximately 80% of discarded textiles end up in landfills or are incinerated, and less than 1% are recycled back into textile fibres (Shirvanimoghaddam et al., 2020). The generation of post-consumer textile waste (PCTW) is particularly high in developed nations, with per capita figures reaching 41 kg in Australia, 38.7 kg in the United States of America (Crestani et al., 2023) and 16 kg in Europe annually (DeVoy et al., 2021; Juanga-Labayen et al., 2022). These countries often rely on incineration or export their waste to offshore destinations. For instance, the European Union (EU) exported around 1.7 million tons of its 5.8 million tons of annual textile waste to Africa and Asia in 2022 (Crestani et al., 2023). Similarly, Australia shipped a significant portion of its 210,000 tons of PCTW in 2023 to the United Arab Emirates and Malaysia (DCCEEW, 2022; Du et al., 2023; Nina Gbor, 2024).

The current practices highlight disparities in global textile waste management, calling for integrated technological advancements and policy frameworks that promote environmental sustainability through enhanced domestic waste management infrastructure.

### Significance of waste management in the textile industry

PCTW management often includes only recycling, but for improved sustainability, this industry needs to aim towards a circular economy, which includes reduction, reuse, recycling and energy recovery (Yalcin Enis et al., 2019). Circular activities can be applied across the textile life cycle. In raw material production, sourcing sustainable and durable materials reduces environmental impact. During manufacturing, precision processes and design for durability, repairability and long life can minimize waste, with artificial intelligence offering the potential for optimized resource use. For example, 12–30% of raw materials are currently wasted during the spinning, knitting/weaving, wet processing, cut, make and trim process, which highlights the need for efficiency improvements (Khairul Akter et al., 2022). Reuse, which extends the life of unsold garments and PCTW, has a significantly lower environmental footprint, reducing carbon emissions by an estimated 3 kg of CO_2_ for every medium-to-high-quality clothing item reused (Nina Gbor, 2024). Approximately 45% of PCTW is reusable. Recycling, which involves repurposing fibres, can potentially address 70% of PCTW (Shirvanimoghaddam et al., 2020). Energy recovery offers a solution for the 10% of non-recyclable textiles, which can be incinerated to generate energy. Adopting circular practices across the life cycle stages is essential to create a sustainable textile industry. Hence, adopting a circular textile waste management strategy in the textile industry helps mitigate the industry’s overall environmental impact.

### Role and types of governmental initiatives

Given the complexity, textile waste management requires the active participation of multiple stakeholders, including government bodies, recycling industries, logistics providers, research organizations, NGOs and consumers. The government plays a pivotal role in shifting the textile industry from a conventional linear model to a more circular one by coordinating these stakeholders, fostering collaboration and creating the necessary infrastructure to support effective waste management. These can be categorized into action plans and strategies, policies, regulations and laws, financial incentives, education and awareness programs, and setting targets and roadmaps. Notable examples include the European Commission’s (EC) adoption of the Waste Framework Directive (WFD) and the Australian government’s efforts to foster domestic recycling through clothing stewardship programs (Bukhari et al., 2018; Khan et al., 2023). Such initiatives aim to enhance waste management, create local employment opportunities and drive sustainable practices within the textile sector.

### Objective of the review

Previous studies have addressed aspects of textile and circular economy policy, contributing insights that this review builds upon and addresses. Payne et al. (2022) provided a valuable global scan of national textile policies, offering a descriptive mapping that sets the stage for a structured, criteria-based assessment or deeper analysis of regional implementation dynamics. Milios (2018) offered a systems-level evaluation of EU resource efficiency policies through a life cycle lens, identifying underutilized policy areas and advocating integrated policy mixes, providing a foundation for the analysis tailored to the specific complexities of textiles. More recently, Puglia et al. (2024) introduced the Circular Policy Canvas to map EU fashion textile industry policies across six CE-specific dimensions, delivering a powerful visual and methodological tool, creating opportunities to explore cross-regional policy transferability or national-level divergence. Building on these contributions, this review integrates the sector-specific focus with the systems-level approach and the global perspective, applying a structured evaluation of textile waste policies across three analytical dimensions – lifecycle stage targeted, breadth of scope, and implementation intensity – across multiple world regions. By critically comparing regional policy frameworks and examining their effectiveness, this study offers not only a comprehensive mapping of the global textile policy landscape but also an evidence-based foundation for identifying transferable best practices and closing lifecycle governance gaps.

## Methodology

### Literature review

Several studies have assessed policies or policy-related drivers for circular economy transitions, though most are either sector-specific or regionally limited. Milios (2018) examined EU resource efficiency policies using a life cycle framework, identifying underutilized areas such as reuse/repair/remanufacturing, green public procurement and secondary materials markets, but without focusing specifically on textiles. Puglia et al. (2024) developed the Circular Policy Canvas to map EU fashion textile industry policies across six CE dimensions, revealing lifecycle imbalances and a lack of inner-loop strategies, yet confined to EU-level scope. Other works, such as Brydges (2021) and Ghisellini et al. (2016), explore policy-related barriers, governance fragmentation and the importance of coherent policy mixes, while Ghisellini et al. (2016) compare CE adoption across regions to highlight political and cultural drivers.

Among these, only Payne et al. (2022) provide a comprehensive global scan of national textile policies, mapping diverse interventions such as Extended Producer Responsibility (EPR), waste bans, repair incentives and recycling targets. Building on this foundation, the present analysis adopts a primarily qualitative, ex-ante approach – summarizing existing data and offering recommendations rather than quantifying policy impacts, as many initiatives are recent (post-2020) and influenced by external factors beyond this study’s scope.

A two-stage process was employed: first, compiling a comprehensive list of governmental initiatives across regions (Table 1) based on Payne et al.’s global scan; second, expanding this through systematic web searches and government website analysis, guided by the keywords identified in Payne et al. (2022). Upon qualitatively screening the global scan report of Payne et al. (2022) the regional policy documents cited within the report, the following keywords were identified for use in further searches: (‘textile OR clothing OR garment OR apparel AND waste’) AND (‘policy’ OR ‘framework’ OR ‘laws’ OR ‘Extended Producer Responsibility’ (EPR) OR ‘Textile Recycling Targets’ OR ‘Tax Incentives’ OR ‘Subsidies,’ ‘waste reduction regulations’ OR ‘import and export regulations on textile waste’ OR ‘recycling policy’ OR ‘circular economy’ OR ‘reuse programs’ OR ‘lifecycle analysis’ OR ‘recycled content’ OR ‘repair incentives’ OR ‘waste bans’ AND/OR (‘Country name’). These keywords and their combinations were used to perform a grey literature survey through Google Search, enabling the identification of additional documents and initiatives for analysis. The review was stopped after November 2024, so the latest developments may not be evident here. This review compiles policy measures addressing textile waste management, examining approaches adopted by various countries, identifying regions with prevalent measures and highlighting common practices. Given the fragmented nature of available information, this review aims to provide a broad perspective but is not exhaustive of all existing mechanisms at the time of writing.

**Table 1.** Regional initiatives identified from the National Clothing Product Stewardship Scheme design document (Adopted from Payne et al. (2022)).

Region	Initiative	Reference
France	Extended Producer Responsibility	(Ellen Macarthur Foundation, 2024)
Sweden	Extended Producer Responsibility	(Brinkmann, 2023)
UK	Voluntary Stewardship	(Payne et al., 2022)
UK	Sustainable Clothing Action Plan	(Ecotextile, 2024)
EU	Circular Economy Action Plan	(CEAP, 2024)
EU	Waste Framework Directive	(European Commission, 2024)
EU	Waste Shipment Regulation	(The European Parliament and of the Council, 2024a)
EU	Ecolabel for textile products	(Ecolabel, 2014)
EU	Registration, Evaluation, Authorization, and Restriction of Chemicals (REACH) Regulation	(European Commission, 2007)

Policies evaluated by us include regulatory measures, voluntary instruments, road maps and financial support focused on textile waste management. The study excludes draft proposals, policies for allied industries (like sustainable raw material production or dyeing), industry-led initiatives and broader environmental frameworks that exclude textile waste management milestones. The initiatives from the following levels of administration were considered: city/council/local government, state/province/territory and national and regional levels.

In this review, ‘textile waste’ encompasses clean, pre-consumer and post-consumer textile waste. ‘Textile waste management’ refers to reducing textile waste at production, processing and post-consumer stages through process optimization, consumer awareness, slow fashion, product life extension strategies (like reuse, repair and resell), prevention of landfilling and recycling. The textile waste management approach begins by optimizing raw material processing to reduce waste during the cut, make and trim phase, followed by designing textiles for longevity, repairability and reusability. It further emphasizes refining the waste management hierarchy to minimize landfilling and enhance closed-loop recycling.

Given the varying roles countries play in the textile supply chain – as producers, consumers or both – the study identifies the specific lifecycle stages each policy targets. Further, the analysis focuses on policy documents available in English through the government’s website.

### Classification and analysis of policies

The review followed a comparative analysis to organize and evaluate initiatives by tabulating descriptive information of policy frameworks and assessing available progress reports, as well as performance data (Skillington et al., 2022; Ürge-Vorsatz et al., 2007).

Table 2 provides categories of initiatives, and Table 3 provides attributes of initiatives considered in the study. We also included government-led awareness programs, recognizing their role in raising consumer awareness about sustainable consumption and waste management. The analysis focused on initiatives defined by their objectives (ends) and mechanisms (means) (Howlett & Cashore, 2009; Skillington et al., 2022). It differentiated initiatives based on effectiveness and signs of change, considering two dimensions: (1) initiative density (number of policies in each country) and (2) initiative intensity, which was further divided into target intensity (specific goals and reduction of waste) and scope intensity (lifecycle stages covered, population engagement and scale of implementation), as detailed in Table 4 (Howlett & Cashore, 2009; Skillington et al., 2022).

**Table 2.** Description of the initiative types considered for the study.

Initiative	Definition
Frameworks/roadmaps	Strategic guidelines and action plans that outline textile waste management objectives and set future targets, often serving as precursors to regulatory measures aimed at achieving these goals over time.
Regulatory Measures	Enforceable legal instruments – including laws, regulations and official policies – that require stakeholders to comply with standards. These measures often mandate or incentivize actions related to reuse, repair, recycle and ecodesign.
Voluntary Instruments	Diverse, voluntary and non-binding tools aimed at fostering behaviour change, knowledge sharing and innovation. These include government-led voluntary programs, such as stewardship initiatives, production guidelines and procurement standards.
Financial Support	Providing financial assistance to strengthen infrastructure and build capacity for enhanced textile recycling systems.

**Table 3.** Attributes of initiatives considered in the study.

Attribute	Description
Name	Name of the initiative examined.
Date	States the year of implementation,
Stage of Life Cycle Addressed	It highlights the lifecycle stage (e.g., design, use phase, end-of-life) targeted, ensuring alignment between the initiative and textile waste management goals.
Type	Categorizes the initiative (e.g., regulatory, voluntary, financial) as per classification in Table 1, aiding in comparative analysis across different policy types.
Administrator / Beneficiary Organization	Identifies the organization responsible for the initiative’s administration, showing stakeholder roles in policy execution.
Entity type	Specifies whether the body responsible is a government, private entity or public-private partnership, indicating the nature of stakeholder involvement.
Scale	Indicates the geographic scope (local, state, national), clarifying the initiative’s area of influence.
Typology	Describes the stakeholders or target groups involved, helping to understand who is responsible for or impacted by the initiative.
Target	Defines the main objective (e.g., waste reduction, recycling increase), providing clear goals to measure the initiative’s success.

**Table 4.** Grading scale used for qualitative assessment of policy intensity.

Category	Low (Low)	Medium (Med.)	High (Hi)
Intensity – Targets	Minimal or no defined goals or milestones; guidelines only; or limited actions like awareness campaigns or small-scale collection.	Moderate targets include up to 25% waste reduction and some infrastructure for collection without comprehensive milestones.	Ambitious goals, including large-scale recycling infrastructure, ecodesign or national waste reduction targets.
Intensity – Scope	Narrow focus (e.g., consumer awareness), small-scale application (e.g., city-level), limited material types or stakeholders.	Broader collection and partial recycling coverage applies to more regions or sectors but do not fully address the entire lifecycle.	Comprehensive coverage of multiple lifecycle stages involving diverse stakeholders and nationwide application.

The methodology recognizes that the number of initiatives alone does not indicate effectiveness. For example, a country with a few high-intensity policies can perform better than one with numerous low-intensity, localized voluntary schemes. Therefore, both density and intensity were evaluated concurrently to avoid misrepresenting progress. Although the study did not employ a formal triangulation method such as stakeholder interviews, the use of diverse data sources – including government websites, academic literature and policy databases – served to reduce interpretive bias. Moreover, policy categorization was internally cross-validated to ensure consistent classification across regions.

## Regional distribution of initiatives for textile waste management

We evaluated textile waste management strategies in five regions – Oceania, Europe, Asia, North America and Africa. South America was excluded due to the absence of any regional incentives at the time of writing. Due to uneven policy transparency and documentation across regions – particularly in parts of Africa, Asia, Latin America and Oceania (excluding Australia) – the comparative depth of analysis may vary depending on the availability and accessibility of government-released data.

### Oceania

Australia has taken the most significant steps toward managing textile waste among Oceania countries. As a major consumer nation, it imported 97.39% of its clothing in 2018‒2019, with around 2% produced domestically (Payne et al., 2022). Australians are the largest per capita consumers of textiles, purchasing 27 kg of clothing textiles annually and generating 23 kg of textile waste per person (Khan et al., 2023). Half of this waste is landfilled domestically, with a quarter exported, often ending up in overseas landfills, while less than 10% is recycled or reused (Australian Fashion Council, 2023). The issue of textile waste gained significant attention in Australia in 2020, prompting the government to take its first major step by organizing a roundtable in 2021 to discuss potential measures and develop an action plan to mitigate its impact (Piller, 2022). Since then, a series of national, state and council-level initiatives have been launched, as detailed in Table S.1.

The ‘Seamless: National Clothing Product Stewardship Scheme’ or NCPS Scheme (Seamless Report, 2023) is a promising initiative to tackle textile waste through a voluntary 4-cent levy on clothing products. The NCPS scheme covers multiple aspects, including circular textile economy, eco-modulation and circular business models (CBMs), while promoting behaviour change through consumer education (Seamless Report, 2023). The NCPS aims to maximize clothing use through a circular textile economy by adopting R-strategies, including refuse, rethink, reduce, reuse, repair, refurbish, remanufacture, repurpose, recycle and recover. Eco-modulation is a criterion for reducing levies on clothing materials and applies to garments containing at least 95% mono-material or single fibre as the primary material. Typically, CBMs promote a circular textile economy by using sustainable, longer-lasting clothing materials, and these companies are genuinely committed to R-strategies in the long run.

The expected turnover of the NCPS is estimated to range from approximately $23 million to USD 39 million (36 million to 60 million AUD). This revenue will support various initiatives, including establishing a monitoring body for sustainable manufacturing and the clothing industry, expanding recycling facilities and enhancing consumer engagement (Seamless Report, 2023). The participating industry receives exclusive perks, including a 25% eco-modulation discount for sustainable products. Seamless could help build consumer trust by showcasing a brand’s commitment to environmental responsibility and also keep brands compliant with evolving environmental regulations, reducing risk and positioning them for long-term success.

However, its scope is limited to clothing products, which represent only 60% of total textile waste in Australia (Nina Gbor, 2024). For example, between 2015 and 2019, textile imports to the state of New South Wales alone comprised 43% clothing, 26% furnishings, 15% raw textiles and 10.9% carpets (NSW EPA, 2021). Moreover, the scheme’s voluntary nature and target of involving only 60–80% of stakeholders create a risk of non-participants benefiting without contributing. Additionally, the authors find a lack of representation of research institutions and environmental scientists in the NCPS scheme’s advisory group. This absence limits the inclusion of scientific insights that could enhance decision-making within the stewardship scheme.

The state of Victoria in Australia provided a 2.6 million USD (4 million AUD) fund to expand the private facility for accrediting and monitoring textile businesses for ethical manufacturing, which is a positive step in addressing production practices within Australia. However, this initiative primarily addresses domestic practices, overlooking the larger issue of imported textiles dominating the Australian market. This initiative holds the potential for integrating with the NCPS Scheme to develop stronger regulations for circular textile design.

The National Environmentally Sustainable Procurement Policy of Australia, or NESPP, is an initiative designed to ensure that materials and services procured by the government are sustainably and ethically produced. The scheme has a broader scope, covering many textiles beyond clothing. However, the policy’s impact is diluted by its minimum purchase threshold of 650,000 USD (1 million AUD), which allows smaller tenders to bypass ecodesign requirements. This creates a gap in ensuring that all textile-related purchases meet sustainability standards, reducing the policy’s overall effectiveness.

Australia’s approach to textile waste management stands out due to its multi-tiered governance structure, involving stakeholders from national to council levels. Including various states, entities demonstrate a highly responsive and climate-conscious governance model that promotes the development of a circular textile industry. Most of the government’s initiatives are focused on financial support, enhancing recycling and sorting infrastructure.

Despite the intense focus on infrastructure development in Australia, summarized in Table 5, the intensity of its targets remains low due to several gaps. First, the regulations are not mandatory and are limited in scope. Second, the stewardship program covers only clothing textiles, leaving out other products, such as footwear, upholstery and unfinished textile waste (Seamless Report, 2023). This narrow focus limits the overall effectiveness of the initiative. Third, over a quarter of the total funding allocated across national, state and council levels for recycling infrastructure has been concentrated on one recycling entity, Blocktexx, which restricts broader distribution and inclusivity across the different initiatives. This concentration of resources raises concerns about the equitable allocation of support, as other areas of textile waste management may not receive sufficient investment to innovate and grow. Addressing these issues, ensuring equitable resource distribution and including scientific expertise in decision-making will be crucial for advancing circular textile management in Australia.

**Table 5.** Overview of textile waste management initiatives in Australia: Policy types, coverage and key challenges.

Attribute	Summary
Dominant Policy Types	Voluntary + Financial Support + Regulatory (e.g., Seamless scheme, federal and state-level grants, procurement frameworks)
Average Intensity Level	Medium-High
Lifecycle Coverage	Design, Manufacturing, End-of-Life
Enforcement Mechanisms	Primarily voluntary, though procurement policy mandates apply to public institutions
Funding Support	High – Federal and state grants, support for infrastructure (e.g., sorting hubs, recycling facility), public procurement mandates
Major Gaps / Challenges	Voluntary nature limits enforceability; slow adoption among smaller producers; no national EPR mandate; Lack of researchers representative in the NCPS board.

### Europe

#### Initiatives undertaken by the European Commission

The EU member states operate under a two-stage legislative process. The EC is the executive body that proposes directives, regulations and frameworks. Once these proposals are put forward, they undergo a rigorous review and negotiation process within the European Parliament and the Council of the EU, which comprises member states’ representatives. EU directives require member state to transpose the provisions into national law, allowing flexibility in implementation. EU regulations are directly applicable across all member states without the need for national transposition. However, individual member states can also promote textile circularity proactively. For instance, France, Latvia and the Netherlands have all implemented an extended producer responsibility (EPR) scheme before EU-wide policies (OECD Library, 2016).

Like Australia, the EU textile sector is primarily consumer-driven, with each person consuming an average of 26 kg of clothing and textiles and generating 16 kg of clothing waste annually. Only 28–35% of the EU’s textile demand is met by domestic production. Consumer use accounts for 82% of textile waste, the remaining from industrial, manufacturing or unsold items (Sanjrani et al., 2024). Approximately one-quarter of this waste (4.4 kg per capita) is collected separately for reuse and recycling, while the bulk is disposed of in mixed household waste (Shahid et al., 2024). Notably, 25% of all collected textile waste is exported, with 46% of used textiles sent to Africa and 41% to Asia in 2022 (Solis et al., 2024). Despite this reliance on imports, the EC has implemented measures with high-intensity scope and targets to promote sustainability and circularity within the textile industry across the lifecycle, mainly through its EU Strategy for Sustainable and Circular Textiles, as summarized in Table 6 and detailed in Table S.2 in the supplemental file. This strategy addresses all stages of the textile lifecycle – from production to end-of-life – aiming to reduce textile waste, promote circular design, decrease emissions beyond European borders and promote domestic textile waste management.

**Table 6.** Overview of textile waste management initiatives in the EU: Policy types, coverage and key challenges.

Attribute	Summary
Dominant Policy Types	Regulatory + Strategic Frameworks (e.g., ESPR, EU Textile Strategy, mandatory separate collection by 2025)
Average Intensity Level	High
Lifecycle Coverage	All stages – Design, Use, End-of-Life
Enforcement Mechanisms	Partially – mandates (e.g., waste collection) are binding; other elements (like ecodesign requirements) are pending full implementation
Funding Support	Moderate
Major Gaps / Challenges	No mandatory EPR across all Member States; fragmented implementation; complex compliance environment for SMEs; textile waste infrastructure development uneven

Four notable initiatives discussed in this review are the Ecodesign for Sustainable Products Regulation (El Darai et al., 2024) to promote design for circularity, the Unfair Commercial Practices Directive (UCPD) to improve transparency for consumers, the WFD to introduce EPR and the Regulation on the Registration, Evaluation, Authorization, and Restriction of Chemicals (REACH).

ESPR applies to all physical products except food, feed and medicinal products. It establishes a regulatory framework reinforcing ecodesign requirements, emphasizing durability, circularity, repairability and recyclability. For textiles, the framework explicitly targets improvements in these areas to reduce consumption-related emissions and minimize the overall environmental footprint within the EU. Key provisions include a ban on the destruction of unsold goods, introducing a digital product passport (DPP) for product transparency, focusing on green public procurement (GPP) and encouraging sustainable product choices in public sector tenders. The DPP mandates a unique identifier recording compliance details, substances of concern, user manuals, safety instructions and disposal guidance. GPP emphasizes that all government purchases, including goods, services and works, should prioritize sustainability and have reduced environmental impacts throughout their lifecycle compared to alternatives with the same function. Further details are provided in Table S.2. These measures aim to reduce waste, lower emissions and promote circularity throughout the textile lifecycle (Nickel, 2024; The European Parliament and of the Council, 2024b). The EC will adopt the ESPR work plan in the first half of 2025, with a detailed prioritization of product categories. Following this, the gradual rollout of product-specific regulations is set to begin from 2026 onwards.

The UCPD protects consumers from misleading practices, focusing on areas like environmental claims, planned obsolescence, dual-quality marketing and transparency in online platforms. In February 2024, an amendment was adopted to empower consumers for a green transition, with implementation set for September 2026. This amendment enhances consumer protection by requiring brands to disclose product durability, repairability and recyclability, curbing false greenwashing claims. Enforcing transparency empowers consumers to make sustainable choices, pushing brands towards more ethical and environmentally friendly practices.

The most significant initiative for textile waste management within the EU is the proposed amendment to the WFD. The amended WFD mandates the implementation of EPR schemes for textiles across all EU member states. The existing WFD already requires the separate collection of textile waste starting in 2025, and this new amendment will further drive infrastructure development for efficient textile recycling. Currently, 2.1 million tons of post-consumer textile waste are collected annually for recycling or reuse, representing 38% of the market, while 62% are discarded (Nørup et al., 2019). A key component of the WFD is EPR, a mechanism that holds producers accountable for covering waste management costs. The newly proposed EPR regulations incorporate an eco-modulated fee to enhance textile waste collection, sorting, reuse and recycling while promoting circular design practices. This fee, paid by producers placing products on the market, supports the development of infrastructure for textile collection, sorting and recycling. EPR will help prioritize waste prevention and repair, which can further reduce the overall volume of textile waste generated.

Under the WFD, a new regulation on textile waste export limits the export of such waste to non-OECD countries. This measure ensures that textile waste is managed within the EU, prioritizing recycling and reuse (The European Parliament and of the Council, 2024a). This policy drives governments to improve domestic collection and recycling infrastructure, reducing the environmental damage caused by inadequate waste management practices in non-OECD countries. Awareness programs, such as the EU’s ‘Empowering Consumers for the Green Transition’ Directive, help educate consumers on sustainable choices. Though less intense, these programs are vital in shaping consumer behaviour and supporting broader circular economy goals.

The EU’s REACH policy came into effect on June 1, 2007, and is a key framework addressing the environmental impact of recycling industries. It ensures that chemical emissions from these industries are appropriately managed to prevent ecological harm (European Commission, 2007). It has since been amended multiple times to include stricter rules on safety data sheets, limit the use of harmful chemicals, restrict PFAS substances and is currently undergoing further amendments to align with the European Green Deal and the Chemicals Strategy for Sustainability. REACH protects human health and the environment from chemical risks while boosting the EU chemicals industry’s competitiveness. It covers substances in industrial processes and everyday items like cleaning products, clothing and electronics. Companies manufacturing or importing over 1 ton of chemicals annually must register with the European Chemicals Agency (ECHA), proving safe use and managing risks. If risks are unmanageable, substances may be restricted or banned. REACH also promotes safer alternatives to hazardous chemicals. It is especially crucial for the textile recycling industry, where high chemical use can release toxic derivatives, organic contaminants and microplastics, ensuring recycling processes are safer and more sustainable (Ruiz-Caldas et al., 2024).

#### Individual initiatives by European countries

Several European countries have made significant progress in addressing textile waste through various innovative policies, regulations and initiatives, listed in Tables S.3 to S.11. Table 7 summarizes the key features of initiatives across Europe. While France stands out for its comprehensive national initiatives, other countries have either focused on a single stage of the textile lifecycle, such as waste collection, or have only recently implemented policies, like Latvia’s EPR scheme, which still requires stronger direction in eco-modulation and infrastructural support. While these measures vary in scope and focus, the cumulative effect has been a steady move towards circularity in the textile sector. By implementing EPR schemes, incentivizing repairs and mandating separate textile collection systems, many European countries have demonstrated that a well-structured approach to textile waste management can lead to substantial environmental and economic benefits, as shown in this section.

**Table 7.** Overview of textile waste management initiatives across European countries and France: Policy types, coverage and key challenges.

Attribute	Summary - Europe	Summary - France
Dominant Policy Types	Regulatory + Strategic Frameworks +EPR (Implemented in few countries like France and Latvia)	Regulatory + Economic (EPR, AGEC Law, Repair Bonus)
Average Intensity Level	Medium, and uneven – some countries (e.g., France, Germany) show mature implementation, while others are still adopting baseline compliance measures	High
Lifecycle Coverage	All stages yet not uniformly enforced	Design, Use, End-of-Life
Enforcement Mechanisms	Partially enforced: mandatory collection laws are binding; eco-modulation, durability labelling and EPR frameworks vary widely by country	Yes – legal penalties for non-compliance (e.g., ban on destroying unsold goods)
Funding Support	Moderate	Moderate to High – direct repair subsidies, eco-modulation incentives via EPR
Major Gaps / Challenges	- No mandatory EPR across all Member States- Fragmented rollout of policies- SMEs face regulatory complexity- Infrastructure gaps	Despite enforcing mandatory regulation for long time, progress is slow

France has pioneered EPR for textiles since 2007 and other textile waste management laws, as listed in Table S.3 and summarized in Table 7 (OECD Library, 2016; Wilson, 2021). Under the EPR scheme, manufacturers, distributors and importers are accountable for managing the end-of-life of their products. The system includes eco-modulation and incentivizing circular textile practices by adjusting fees based on product durability and recyclability. The organization Refashion, an eco-organization approved by public authorities and funded through contributions from its members, oversees EPR compliance in France. This scheme has notably increased textile collection rates, driving brands towards more sustainable practices, especially with recent amendments promoting recycled materials. As of 2021, 34% of garments sold in France were collected through the EPR program, with 81% of items being eco-modulated based on durability, generating 53.8 million USD (51.1 million EUR) in fees. Refashion supports registered sorters by paying 84.3 USD (80 EUR)/ ton for items sorted for reuse and 189 USD (180 EUR)/ ton for those sorted for recycling or solid recovered fuel. The EPR initiative has significantly boosted textile recovery, with 56% of collected textiles being reusable and 32% recyclable (OECD Library, 2016; Puglia et al., 2024). Additionally, in 2020, French sorters/ exporters sold sorted clothing to Ghanaian importers for an average of 0.72 USD per kg (Choat, 2023).

The Anti-Waste and Circular Economy Law (AGEC) of France, enacted in 2020, broadly targets to phase out single-use plastics, eliminate waste, enhance resource management and provide transparent information for consumers (Ellen Macarthur Foundation, 2022). Specifically for the textile industry, the law focuses on minimizing waste throughout the textile life cycle and increasing the use of recycled and sustainable materials. Companies placing new products in the French market are required to publish detailed product sheets outlining the traceability of materials, the release of plastic microfibers, the incorporation of recycled materials, the presence of hazardous substances, recyclability and possibilities for reuse (Bcome, 2020; Ellen Macarthur Foundation, 2022). The AGEC Law also adopts specific measures from EPR schemes, requiring companies to manage the end-of-life of their products by joining eco-organizations and paying an eco-contribution for waste management (Article 62). It includes a bonus-malus system where companies are rewarded or penalized based on their products’ environmental performance (Article 13). Transparency is ensured by mandating companies to inform consumers about these bonuses or penalties, while eco-organizations must develop sorting logos to guide proper disposal (Article 17). The law also prohibits the destruction of unsold goods, instead encouraging donation or recycling per the waste treatment hierarchy, with exceptions made for hygiene and childcare products (Rödig et al., 2021). Additionally, incentives for repairs have been introduced, allowing consumers to claim between 6.2 USD (6 EUR) and 26.3 USD (25 EUR) for repairing clothes and shoes at approved workshops. This repair incentive, supported by a 162 million USD (154 million EUR) government fund, is designed to extend the life of products and reduce waste (Bajaj, 2023). France’s comprehensive approach to textile waste management has been highly effective. The latest amendment of EPR in November 2022 encourages brands to integrate recycled materials, adopt environmental labels and enhance the durability of products, setting ambitious targets for 60% textile waste collection by 2028 and 70–80% recycling by 2027 (Cimetiere, 2023).

The Netherlands is amongst the top ten global exporters of clothing waste, but has introduced an ambitious EPR scheme to enhance textile circularity. This initiative targets a 50% rate of textile recycling or reuse by 2025, with fibre-to-fibre recycling goals set at 25% by 2025 and rising to 33% by 2030 (Government of the Netherlands, 2024). Currently, less than 5% of textiles in the Netherlands are recycled, with only 1% undergoing closed-loop recycling. The proposed EPR framework, with its emphasis on textile-to-textile recycling, is expected to drive the development of infrastructure and establish a robust recycling system within the Netherlands. Latvia has also implemented an EPR scheme for textiles, requiring producers to take responsibility for the end-of-life management of textiles and apparel. Combined with its existing waste prevention program, Latvia’s approach to achieving complete resource recovery in the textile industry, alongside other waste streams, shows significant potential (Ministry of Smart Administration and Regional Development Republic of Latvia, 2021).

Germany is one of the largest global exporters of textiles and fashion, with European countries serving as significant destinations for German clothing exports. Approximately 50% of its production demand is fulfilled domestically, with 43% sourced from offshore production. The country benefits from a robust infrastructure for structured textile waste collection and reuse, capable of collecting and sorting nearly 75% of its generated textile waste (Première Vision Paris, 2024; Deutsche Recycling, 2023). Germany’s revised Circular Economy Law has introduced strict regulations to prevent the destruction of overproduced, unsold and returned products. This includes implementing a duty of care (Obhutspflicht) and a reporting obligation (Berichtspflicht) for all products (European Environmental Bureau, 2023). The duty of care obligates companies to responsibly manage products throughout their lifecycle, prioritizing reuse, recycling or proper disposal to reduce waste (Rödig et al., 2021). However, this approach is considered to have a medium-intensity scope because the proposed law does not restrict the export of textile waste for destruction, leaving the loop incomplete.

Through its Environment Agency, Norway has mandated separate textile waste collection starting January 1, 2025, to align with EU waste management targets. This regulation aims to raise the current reuse and recycling rate from 37% to 65% for household and similar business waste by 2035 (Interior Daily, 2024). Several EU countries have also introduced repair incentives to promote textile reuse. For instance, Belgium reduced its value-added tax (VAT) to 6% for repairs in clothing and shoes, while countries like Ireland, Luxembourg and Poland introduced similar VAT reductions or mandates for spare parts availability (Bajaj, 2023; ECC-NET, 2024; Marosa, 2023; Ville De Luxembourg, 2022).

The United Kingdom also has a detailed textile waste management plan that promotes circularity and reduces environmental impact through voluntary initiatives and research programs, listed in Supplemental Table S.7. The Textiles 2030 program covers over 62% of the market. It aims to cut the sector’s carbon and water footprints by 50% and 30%, respectively, by 2030, building on the success of the earlier Sustainable Clothing Action Plan (Department for Environment Food & Rural Affairs, 2023; Millward-Hopkins et al., 2023). In parallel, the UK is piloting industry-initiated voluntary EPR schemes to shift the burden of textile waste management to producers. The Circular Fashion Program, with 19 million USD (15 million GBP) in funding, supports innovation and scaling of CBMs, targeting sector transformation by 2032 (Department for Environment Food & Rural Affairs, 2023). Additionally, the UK is developing a Textile Waste Hierarchy, which includes measures like mandatory textile collections and banning textile disposal in landfills. The Interdisciplinary Textiles Circularity Centre, backed by 6.8 million USD (5.4 million GBP), focuses on research into renewable textile materials from post-consumer and household waste (Department for Environment Food & Rural Affairs, 2023; Millward-Hopkins et al., 2023).

These targeted initiatives have driven substantial progress, positioning several countries above the EU average. In France, EPR regulations have resulted in a textile waste collection rate of 38%, well above the EU average of 12%, supported by the establishment of approximately 44,000 textile and footwear collection points, equating to one per 1,490 people (European Environment Agency, 2024). Other countries with robust textile collection systems have also demonstrated strong performance, with Germany (62%), Luxembourg (50%), Belgium (50%), the Netherlands (37%) and Austria (30%) leading the way (Duiker, 2024; European Environment Agency, 2024).

Significant challenges persist while these initiatives demonstrate a structured approach to targeted waste reduction and enhancing circularity within the textile industry. Despite achieving high collection rates, the lack of adequate domestic recycling infrastructure in countries like Germany and France has resulted in the export of a substantial portion of collected textile waste (The Brussels Times with Belga, 2024). In 2023, 60% of textile waste collected in Germany and over 80% in France in 2021 was exported predominantly to countries in the Global South (Duiker, 2024; Ellen Macarthur Foundation, 2024; European Environment Agency, 2024). Although strict regulations mandate textile sorting and collection, the inability to process this waste locally leads to mismanagement and a reliance on exports.

The regulatory complexity and varying adoption rates in European countries make it challenging for producers to navigate Europe’s textile circularity initiatives (Niinimäki et al., 2024). A lack of accessible data regarding material composition and microplastic and chemical release across the textile life cycle hampers transparency, complicates recycling efforts and hinders sustainability assessments (Clark, 2023; Undas et al., 2023). Addressing these issues, ensuring uniform implementation across Europe and supporting producers will be crucial for achieving long-term textile circularity in Europe.

### Asia

Asian countries collectively accounted for 71.5% of the global textile export market in 2022, with a total export value of approximately 223.9 billion USD. China, Vietnam, Türkiye and India’s market share rose steadily from 40% in 2010 to 56.8% in 2022 (Lu, 2024). Tables S.12 to S.16 in the supplemental section list existing policies across various Asian countries for textile waste management. In 2017, China, India and Japan collectively accounted for 47.5% of global apparel consumption, with individual consumption levels of 40 billion, 6 billion and 3.3 billion units, respectively (Fashion United, 2023). Despite Asia being the largest producer and significant consumer of textiles and a major importer of textile waste, the region lacks a comprehensive, dedicated regulatory framework for textile waste management. Instead, the region’s efforts as detailed in Table 8 are fragmented, focusing on import restrictions and few production optimization strategies rather than holistic circular economy models for textiles.

**Table 8.** Summary of textile waste management initiatives across Asia: Policy types, coverage and key challenges.

Attribute	Summary
Dominant Policy Types	Fragmented and reactive - Import bans (e.g., Indonesia), production efficiency goals (e.g., China), recycled content mandates (e.g., Türkiye)
Average Intensity Level	Low: Policies are isolated with limited enforcement or lifecycle coverage
Lifecycle Coverage	No lifecycle coverage strategy
Enforcement Mechanisms	Weak Enforcement and no binding circularity targets
Funding Support	Low: No state investment
Major Gaps/Challenges	- No region-wide circularity roadmap- No mandatory EPR- Focus remains on production optimization, not lifecycle design- Infrastructure and data gaps - the region needs additional focus on clean waste (Industrial) mitigation strategy

The initiatives in Asia generally target the end-of-life stage of textiles, with efforts such as Indonesia’s secondhand clothing (SHC) import ban (2015) or South Korea’s ban on waste textile imports (2022) to safeguard domestic industries. These policies prioritize reducing competition from low-cost imports and optimizing local recycling capacity. Unlike other Asian regions, China began prioritizing sustainability in the late 2000s, implementing significant governmental initiatives across various sectors. However, China’s decision to ban the import of waste plastics, including textile waste, had substantial global repercussions (Lin et al., 2023). Before 2018, China was the primary destination for plastic and material waste, including textile waste worldwide. Following the implementation of this ban, the global trade of plastic waste dropped by 45.5%, while China’s imports decreased by 95.4% relative to baseline levels (Islam & Wang, 2023; Lin et al., 2023; Wen et al., 2021). Although specific or exact amounts of textile waste are not provided, this ban has pushed other global regions to look for domestic waste management solutions, including those for textile waste (Qu et al., 2019).

Further, China has set a target to recycle at least 25% of textile waste by 2025, focusing primarily on pre-consumer waste management. This goal is supported by forthcoming policies, such as the ‘Implementing Opinions on Accelerating the Recycling of Waste Textiles,’ which are designed to achieve this recycling target (Islam & Wang, 2023; Lin et al., 2023; Wen et al., 2021). Despite these measures, initiatives specifically targeting textile waste management remain underdeveloped and lack a focused approach; therefore, efficient recycling of textile waste is still a problem (Islam & Wang, 2023; Lin et al., 2023; Wen et al., 2021). Türkiye is one of the few Asian countries with a forward-looking policy framework focused on integrating recycled materials into textile production. As the seventh-largest textile exporter, with European countries being its primary market, Türkiye must adhere closely to the EU’s guidelines to maintain its market position. The Turkish government has committed to using at least 30% recycled materials in new clothing by 2030, with an overarching goal to reduce the environmental impact of the textile sector by 50% by 2035 (Ministry of Foreign Affairs, 2021). This ambitious plan underscores Türkiye’s intention to move toward a more circular economy by promoting recycled content in its vast textile industry, a notable exception within the region. However, despite these efforts, the overall impact of Türkiye’s initiatives remains moderate.

While Asia dominates the global textile supply chain, producing most of the world’s textiles and serving as a significant consumer market, the region lacks comprehensive frameworks for circular textile waste management. Most efforts are focused on discrete measures like import restrictions, production optimization and isolated strategic plans rather than robust frameworks for circularity. This starkly contrasts the EU, which has developed integrated policies targeting the entire textile lifecycle, including ecodesign, recycling and reuse.

### North America

North America, mainly the USA and Canada, plays a significant role in global textile consumption and waste production. Both countries are major consumers of clothing and producers of textile waste. In the USA, over 98% of apparel sold in retail markets is imported, amounting to 148 billion USD, making it the world’s largest clothing importer. Similarly, Canada consumed 1.3 million tons of apparel in 2021, with nearly all clothing imported. Despite their status as major consumer nations, as summarized in Table 9, there is a noticeable gap in national-level policy addressing textile waste, detailed in Table S.17.

**Table 9.** Summary of textile waste management initiatives across North America: Policy types, coverage and key challenges.

Attribute	Summary
Dominant Policy Types	Emerging: Regulatory + Voluntary Frameworks
Average Intensity Level	Low: Policies are state/province-specific, with limited national-level mandates
Lifecycle Coverage	Mostly End-of-Life: Some measures address reuse, sorting and recycling; design and production stages largely excluded
Enforcement Mechanisms	Partial: U.S. federal oversight is limited – enforcement depends on state/local commitment; Canada’s approach is more coordinated but still evolving
Funding Support	Moderate: Some state-level grants (e.g., California fabric recycling pilot), but lack of stable funding for infrastructure
Major Gaps / Challenges	- No nationwide EPR- Uneven adoption across states/provinces- Lack of lifecycle integration- Weak industry accountability mechanisms

In Canada, no significant national, provincial, or municipal legal initiatives or regulations exist to manage post-consumer textile waste. In 2021, Canada consumed 1.3 million tons of textiles and produced nearly 1.1 million tons of textile waste in 2022. However, less than 1% of textile waste is collected. The absence of structured regulatory frameworks highlights the lack of a coordinated strategy to manage textile waste (Government of Canada, 2024). In contrast, the USA has recently recognized textile waste as an environmental issue. In 2018, the USA was estimated to have produced nearly 17 million tons of textile waste. Of this, approximately 14.7% was recycled, 3.2 million tons were combusted, and 11.3 million tons were landfilled. These figures highlight that resource recovery was a significant pathway for textile recycling in the US during that year (United States Environmental Protection Agency, 2024). Between 2023 and 2024, several states introduced regulations to manage post-consumer textile waste. However, while these states’ targets are high regarding reducing waste, the overall scope remains limited. The initiatives are isolated at the state level, lacking a coordinated national strategy. For example, California’s SB 707 (Responsible Textile Recovery Act) mandates an EPR program for textiles, requiring manufacturers and distributors to create statewide collection, recycling and repair systems by 2026, with full compliance expected by 2030 (California, 2024). However, this is restricted to the state of California only.

Similarly, New York City implemented a textiles separation requirement, mandating that businesses recycle or repurpose textile waste if it constitutes more than 10% of their total waste. The New York Fashion Act, if implemented, will mandate fashion brands with annual revenues over $100 million to report on the environmental and social impact of their products and implement enhanced due diligence procedures (Lüttin, 2024). Massachusetts also introduced a statewide textile disposal ban in 2022 (Massachusetts Department of Environmental Protection, 2022), promoting reuse and recycling through programs like ‘Beyond the Bin.’ While these state-level initiatives in the US mark progress, the lack of federal regulation or coordinated national policy limits their broader impact. Like the European frameworks, a nationwide strategy could significantly enhance the scope and effectiveness of textile waste management in North America. Without comprehensive national policies, the US and Canada continue to lag behind other regions like the EU in achieving circularity in the textile industry.

### Africa

Africa has long struggled with the issue of waste colonialism, where SHC and textile waste are often dumped by high-income countries, undermining local industries. This practice also creates severe environmental burdens on countries because 70–85% of clothes remain unused and end up in landfills due to a lack of infrastructure and policy support as summarized in Table 10, to handle PCTW recycling at the present volumes (Besser, 2021; Tuppens, 2023). Table S.18 provides a comprehensive list of all policies currently implemented within the African Union.

**Table 10.** Summary of textile waste management initiatives across Africa: Policy types, coverage and key challenges.

Attribute	Summary
Dominant Policy Types	Import Restrictions + Informal Measures
Average Intensity Level	Low: Policies are nascent or fragmented, with minimal enforcement capacity
Lifecycle Coverage	Narrow: Focus is primarily on end-of-life through bans; production, design and reuse stages largely unaddressed
Enforcement Mechanisms	Weak: Bans often poorly enforced; lack of infrastructure enables informal dumping and illegal imports
Funding Support	Very Low: No governmental support
Major Gaps / Challenges	- No structured national policies- Limited data transparency- Informal markets dominate- Absence of recycling systems - Should focus of self-reliant textile economy

At the national level, Rwanda took a pioneering step in 2018 by banning SHC imports to protect its domestic textile industry. While the policy boosted local production, it increased clothing costs for consumers. Other countries, including Uganda, Kenya, Tanzania, Burundi and South Sudan, have made similar attempts, though with limited success. Uganda recently banned SHC imports, but the limited domestic textile production infrastructure has made it challenging to meet consumer demand adequately (Gbor, 2020).

African countries have made efforts to implement sustainability broadly in sectors such as manufacturing and the built environment; however, transitioning the existing textile economy toward sustainability has received significantly less attention. Kenya’s Sustainable Waste Management Act (2022) and import regulations enforced by the Kenya Bureau of Standards (KEBS) aim to reduce unwearable SHC imports and promote circular economy principles. Ghana integrates waste management into its Nationally Determined Contributions (NDCs) and SHC inspections, yet recycling facilities remain inadequate. Mozambique’s Green Economy Roadmap (2012) and Green Economy Action Plan (2013) include waste recovery efforts but lack textile-specific strategies. Despite these initiatives, Africa lacks comprehensive frameworks for textile waste management (Oxford Economics, 2024). Policies often emphasize broader waste issues without addressing PCTW specifically. Recycling infrastructure is insufficient, and SHC import bans, while protecting local industries, risk economic strain without parallel investments in domestic production and recycling. Targeted policies and infrastructure development are essential to effectively managing textile waste and supporting sustainable growth.

## Discussion

### Comparative assessment of governmental initiatives

EU and European countries comprehensively address the textile waste issue with medium to high-intensity policies covering all lifecycle stages. Australia stands out for its high policy intensity despite having low policy density. In contrast, Africa, Asia and North America need stronger engagement and more comprehensive policy coverage.

**Figure 1.**
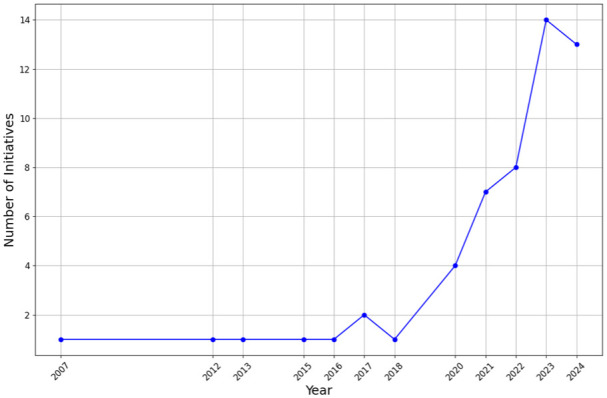
Annual number of textile waste policy initiatives implemented globally until 2024. Note: Data for the year 2024 includes initiatives recorded up to November 2024, in line with the study’s data collection cut-off.

Figure 1 illustrates the distribution of initiatives across the globe over the years, indicating that policy-driven efforts to transition the textile industry toward a circular and sustainable model are a relatively recent development. Notably, 90% of these initiatives were enacted after 2020, highlighting growing momentum but limited evidence of measurable outcomes outside France.

Regulatory measures – such as EPR schemes, waste collection mandates, mandatory recycling laws and product design regulations – remain the most impactful policy instruments for addressing textile waste, particularly when they cover the entire textile life cycle, as demonstrated by France. France has established a high-intensity, high-density framework that, over years of implementation, has developed an efficient waste management infrastructure. Nearly 81% of textiles benefit from eco-modulated fees from EPR, indicating a strong market flow of durable materials. With this foundation in place, France is now focusing on increasing efficiency by raising its targets over the next decade.

Germany, despite fewer measures focused on end-of-life stages, has achieved over 60% textile waste collection, with 75% effectively sorted through mandatory separate collection systems. These examples highlight how mandating regulations can establish strong circular textile systems. Currently, only France has fully implemented policies spanning the entire textile life cycle, while broader EU measures are more recent, with measurable impacts yet to be seen. Financial incentives and support further enhance policy effectiveness, behavioural change and capacity building. VAT reductions on repairs extend product lifespans, while targeted support encourages recycling and the use of durable materials. In Australia, increased government funding from 2020 to 2024 for sorting and recycling infrastructure increased recycling rates by 17%, reduced landfill disposal by 4%, lowered new clothing sales by 12% and boosted second-hand clothing sales by 18% compared to 2018 (Seamless, 2024, 2025). These results show that combining mandatory policies with targeted incentives and capacity-building support improves infrastructure efficiency and waste management outcomes.

Voluntary instruments are common in regions with limited direct government intervention, relying on industry-led initiatives and stewardship programs to promote sustainability. The impact of the Seamless program remains uncertain in Australia in a long run. Its voluntary nature and lack of enforcement may slow adoption, especially compared to France’s compulsory EPR system, which has already delivered measurable improvements in textile collection and recycling rates. Even mandated policies take years to mature, as evident from France, raising doubts about the overall effectiveness of purely voluntary approaches.

Unlike Seamless, which is linked to eco-modulation, CBMs, and covers the entire lifecycle with both short- and long-term goals, the UK’s voluntary approach is more uncertain. The UK’s Textile 2030 program is a high-intensity initiative aiming to reduce carbon and water footprints through circular design and business models, with over 62% market participation. However, it lacks enforceable measures and a clear roadmap for short-term actions, particularly in building recycling infrastructure, nationwide collection systems, eco-modulation incentives and large-scale repair initiatives. This limits its ability to drive systemic change, resulting in low overall policy intensity despite ambitious targets.

In many Asian, African and South American countries, the absence of comprehensive policy instruments limits progress toward circularity. Existing measures are fragmented, weakly monitored and lack coordination, focusing on isolated issues rather than adopting lifecycle-based strategies. For instance, China emphasizes production efficiency, while Türkiye targets 30% recycled content by 2030. Despite these developments, a lack of documented waste management strategies across the life cycle for the textile sector highlights a missed opportunity for reducing significant environmental impacts in these regions (Jordão et al., 2019). Similarly, several nations, such as Denmark, have developed circular economy roadmaps but lack specific tools for textile waste management.

Despite highly intensity and density scope regulatory across Europe to ensure that textile producers are held accountable for the environmental impact of their products the complexity and financial strain involved in adhering to these regulations, particularly in the EU, creates challenges for producers in navigating certification and compliance (Niinimäki et al., 2024). Simplifying these frameworks and improving transparency will be critical to ensuring long-term effectiveness. Awareness programs, though essential, must work alongside more intense measures to ensure a well-rounded approach to textile waste management. They play a key role in consumer engagement but require continuous reinforcement through regulation and economic incentives to drive systemic change.

### Regional scans and suggestions

**Figure 2.**
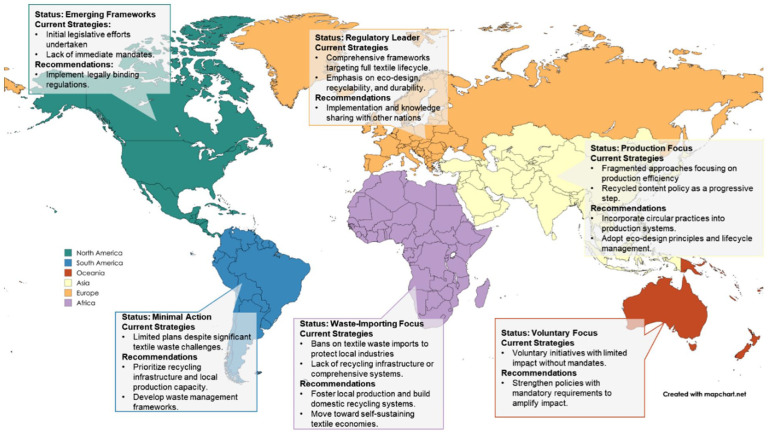
Regional landscape of governmental policy focus and recommendations. Note: Oceania primarily reflects Australia’s contributions, with limited engagement from other nations in the region. Asia, Africa and South America have not taken significant coordinated steps, though each region plays a distinct role within the textile lifecycle – ranging from production to waste import and disposal.

Global response to textile waste management varies significantly across regions (Figure 2), reflecting diverse economic and environmental challenges.

Europe leads, but stronger EU-level alignment, particularly a mandatory EPR – would ensure uniform progress. Additionally simplifying the compliance and certification framework under one roof for EU region in total will help the producers navigate the financial and certification complexities. Australia has made notable strides by investing in domestic infrastructure to support circular textile waste management, offering a model that other developed nations could replicate. However, its voluntary EPR scheme may limit effectiveness, indicating that compulsory implementation is necessary to accelerate impact. Similarly, the UK would benefit from incorporating explicit textile waste management targets, infrastructure development measures, mandatory collection systems and incentives for circular practices to complement its long-term decarbonization goals. Several regions, such as Denmark, have developed ambitious roadmaps for textile waste management; however, delays in implementation may hinder the achievement of their stated targets.

Many initiatives in both the EU and Australia are still recent, and measurable results may take time to materialize. France’s experience demonstrates that a well-enforced regulatory framework, with clear goals and milestones, can progressively build efficient waste collection and sorting systems, offering a model for long-term success in circular textile waste management. Together, the approaches of France, Australia and the EU highlight the importance of combining ecodesign requirements, domestic recyclability, infrastructure building and product durability to advance circularity. These strategies provide valuable insights for other regions, including Canada and the United States, where legislative efforts such as Bill C-337, the Americas Act, frameworks for green procurement and circular textile economies are hindered by the absence of immediate legalization, which delays their potential impact.

Rest of the world lack transparent performance data, preventing the creation of a standardized numerical index without risking misleading interpretations. Producers, region like China, India, Bangladesh and Indonesia, lack comprehensive circular textile waste management frameworks. Türkiye provides an example of progress with its initiative to integrate recycled fibres into production, demonstrating how pre-and post-consumer waste can be addressed through closed-loop recycling. To advance clothing circularity, these regions need clear strategies that optimize production, reduce clean and pre-consumer waste, discourage waste destruction and address end-of-life textiles. This requires robust regulations, infrastructure development, responsible consumption campaigns and measures to prevent illegal waste imports.

African nations, including Rwanda and Uganda, have focused on banning textile waste imports to protect local industries. However, these bans risk straining consumers and failing to deliver sustainable outcomes without adequate recycling infrastructure and waste management systems. Developing domestic production capabilities and recycling systems could enhance economic self-reliance while fostering a circular economy that benefits the environment and local communities. Drawing on successful models from regions like Europe, stricter regulations that hold producers accountable could help integrate ecodesign and green manufacturing practices into these economies (Pérez et al., 2022). To equitably share the burden of post-consumer textile waste, we recommend that revenues generated through a mandated Extended Producer Responsibility (EPR) scheme be partially allocated to support low- and middle-income countries. This funding can be used to build local capacity and develop infrastructure for managing both domestically generated and imported textile waste. Such an approach would promote global fairness and strengthen the collective ability to handle textile waste sustainably.

Although excluded from this review, South America faces significant challenges, with minimal engagement in global textile waste management initiatives. Despite being heavily impacted by textile waste imports, the region lacks structured strategies. Chile, one of the largest importers of second-hand and unsold clothing, is grappling with environmental crises that highlight the urgent need for action (Pérez et al., 2022). Prioritizing infrastructure for textile recycling, expanding local production industries and promoting sustainable practices are critical to achieving long-term environmental sustainability and will also open new markets for material flow and jobs in this region.

Drawing insights from the review, it becomes clear that a multi-tiered approach is crucial for global success in textile circularity. Establishing clear, actionable roadmaps supported by transparent, legally binding regulations is essential to defining producer responsibility, recycling infrastructure and waste management. Regions lagging must focus on fostering local production, developing domestic recycling capabilities and implementing policies to prevent environmental degradation linked to waste imports. Consumer awareness programs must be integrated into these frameworks to encourage sustainable behaviours and active engagement in the circular economy. Balancing regulatory mandates, economic incentives, Eco modulation, state-supported capacity building with industry innovation will ensure transformative progress towards a sustainable and circular global textile industry.

## Conclusion

Policy instruments are essential to driving sustainable and circular textile waste management, especially when compliance is mandatory and enforcement mechanisms are clear. This review shows that mandatory approaches, such as France’s EPR and Germany’s waste collection mandates, have produced more measurable outcomes. Still, challenges remain in expanding coverage to all textile categories, covering all stages of life cycle and ensuring industry-wide compliance.

Government procurement significantly impacts textile flows. Policies requiring sustainable and ethical procurement practices, such as those introduced by the EU and Australia, represent meaningful progress at use and production stage. However, the impact of such policies is constrained by financial thresholds, which permit smaller purchases to bypass sustainability requirements. Removing these thresholds would strengthen policy impact and ensure consistent application.

Despite progress in the EU, implementation challenges remain. Industries face burdensome compliance processes due to complex documentation and economic pressures. Standardizing these requirements across member states, while preserving environmental rigor, would facilitate easier adoption and encourage replication in non-EU contexts. A streamlined compliance architecture can support sustainable textile production and consumption across all supply chain stages.

Producer countries, many of which are also major consumers, need comprehensive policies for sustainable production and circular waste management. These regions generate large quantities of pre-consumer waste, including unsold goods. Therefore, specific policies focused on reducing, reusing and recycling this waste, alongside post-consumer waste, are essential.

In the Global South, textile waste imports have long supported domestic markets but also resulted in severe environmental burdens due to dumping. Some countries have implemented bans on textile waste imports; however, in the long run, policies should focus on sustainable waste management by strengthening domestic production and recycling infrastructure alongside such bans. Without such parallel investment, import bans may simply shift the burden elsewhere rather than creating a long-term solution.

A major policy blind spot globally is the lack of regulation for landfill-bound textiles, which continue to accumulate. To address this issue, cleanup efforts should be supported through pilot-scale projects, large-scale initiatives, voluntary programs, or incentive-based approaches as part of an international organization and a multistakeholder collaborative initiative.

Although international attention to textile waste is increasing, only a handful of countries have adopted comprehensive, lifecycle-based frameworks. Many others are either in early policy development phases or have yet to formally recognize textile waste as a priority. To close this gap, future action must focus on building harmonized international guidelines that support context-sensitive policy adoption across economies at different stages of development.

Investment in circular infrastructure, especially in low- and middle-income countries, will be critical to reducing reliance on imported waste and promoting local recycling ecosystems. Regional repair and recycling hubs, shared technology platforms and cross-border partnerships can further enhance the global textile system’s efficiency and resilience.

## Supplemental Material

sj-docx-1-wmr-10.1177_0734242X251393613 – Supplemental material for Evaluating textile waste management policies: Lifecycle gaps and opportunities for improvement Supplemental material, sj-docx-1-wmr-10.1177_0734242X251393613 for Evaluating textile waste management policies: Lifecycle gaps and opportunities for improvement by Arun Chandra Manivannan, Anubhuti Bhatnagar, Kirsi Niinimäki, Logeshwaran Panneerselvan and Thava Palanisami in Waste Management & Research
