# Supplementary material for: Evaluating textile waste management policies: Lifecycle gaps and opportunities for improvement
Source: Waste Manag Res. 2025 Nov 21;44(5):508–24. doi: 10.1177/0734242X251393613 (PMC13121825; doi:10.1177/0734242X251393613)
Supplement: sj-docx-1-wmr-10.1177_0734242X251393613 – Supplemental material for Evaluating textile waste management policies: Lifecycle gaps and opportunities for improvement [file sj-docx-1-wmr-10.1177_0734242X251393613.docx]

**Evaluating Textile Waste Management Policies: Lifecycle Gaps and Opportunities for Improvement**

Arun Chandra Manivannan^a^, Anubhuti Bhatnagar^b^, Kirsi Niinimäki^b^, Logeshwaran Panneerselvan ^a^, Thava Palanisami ^a*^

1. Environmental Plastic & Innovation Cluster (EPIC), Global Innovative Centre for Advanced Nanomaterials (GICAN), College of Engineering, Science and Environment, The University of Newcastle, NSW 2308, Australia
2. Department of Design, Aalto University, P.O. Box 11000 (Otakaari 1B) FI-00076 AALTO, Finland

*Corresponding author: [thava.palanisami@newcastle.edu.au](mailto:thava.palanisami@newcastle.edu.au)

Supplementary Tables

The tables in this document present the governmental initiatives identified, organized by region, with each row detailing a specific policy and its key elements, while each column represents distinct parameters. These parameters include

1. Name of the initiative
2. Year that the initiative was first implemented or the most recent amendment that expanded its scope to include textile waste management, especially if this is not its primary focus.
3. Description
4. Life cycle stage addressed (from raw material processing to end-of-life)
5. Initiative type (regulatory, voluntary, framework/roadmap, or financial support)
6. Administrator/beneficiary responsible for implementation
7. Nature of undertaking entity (public, private, or public-private partnership)
8. Scale indicating the geographic reach (local, state, or national level)
9. Typology listing stakeholders such as producers, waste management entities, or consumers
10. Target like waste reduction or increased recycling
11. Target intensity measures the ambition of the initiative such low, medium, or high. Low intensity refers to initiatives with limited inclusivity and minimal impact, primarily focusing on awareness. Medium intensity initiatives achieve significant impact but are often restricted to specific regions or groups. High intensity initiatives have a wide reach, addressing multiple lifecycle stages and targeting significant reductions in textile waste on a larger scale.
12. Scope intensity reflects whether an initiative has a narrow (low) or broad (High) approach across various lifecycle stages, such as production, usage, and end-of-life management. Medium scope intensity is attributed due to restricted stage of life cycle addressed or does not promote complete recycling, like addressing only particular stage of life cycle, like collection of textiles.

Table S.1 Summary of governmental initiatives for textile waste management and the stage of life cycle addressed in Australia

| No. | Name | Year | Description | Stage of Life Cycle Addressed | Type | Administrator / Beneficiary Organization | Entity Type | Scale | Typology | Target | Targets Intensity | Scope Intensity | Source |
| --- | --- | --- | --- | --- | --- | --- | --- | --- | --- | --- | --- | --- | --- |
| 1 | Seamless: National Clothing Product Stewardship Scheme | 2023 | 4 cents Levy for all clothing products, reducing the volume of clothing sent to landfills, increasing recycling infrastructure, and incentivizing eco-designs. | End-of-life | Voluntary | Seamless Product Stewardship Organization (Landrigan, Raps et al.) | Public- Private Partnership | National | Textile Producers, Retailers, Waste Management | At least 60% of end-of-life clothing will be reused or recycled by 2027.  Build recycling infrastructure and eco-modulating clothing. | Hi | Med | (Seamless Report 2023) |
| 2 | Federal Government Commercialisation Grant - $1 Million | 2021 | Grant to accelerate the commercialization of textile recycling processes. | End-of-life | Financial Support | Blocktexx | Private Industry | National | Recycling Industry | Upgrade the recycling facility to increase the volume of recycling | Hi | Med | (Australian Government 2021) |
| 3 | National Environmentally Sustainable Procurement Policy | 2024 | The Environmentally Sustainable Procurement (ESP) Policy guides decisions and allows the Government to measure the environmental outcomes from its procurements. Establishes a reporting framework for environmentally sustainable procurement. | Manufacturing and Use Phase | Framework | Government | Government Policy Framework | National | Manufacturers, Procurement Officers, Public Institutions | The government will buy products that: minimize greenhouse gas emissions, are safe for the environment, and retain their value longer. | Hi | Hi | (ESP Policy 2024) |
| 4 | Project Boomerang | 2024 | Investing $4.97 million to support Salvos Stores in establishing a cutting-edge automated sorting hub. | End-of-life | Financial Support | Salvos | Government and NGO Partnership | State | Reuse / Second-hand Clothing Industry | Sort donated textiles to enhance the reuse and recycling rate | Hi | Hi | (Queensland Government 2024) |
| 5 | Victorian Government Fund | 2023 | Allocated $4 million to accredit and monitor textile businesses for ethical manufacturing practices. | Manufacturing | Financial Support | Ethical Clothing Australia | Government-Funded Private Initiative | State | Textile Manufacturers, Ethical Trade Monitoring Bodies | Increase accreditation, monitor more businesses, and provide new educational materials for workplace safety. | Hi | Med | (Victoria 2024) |
| 6 | Resource Recovery Industry Program – $600K | 2024 | Support private clothing waste recycling infrastructure. | End-of-life | Financial Support | Blocktexx | Private Industry | State | Recycling Industry | Support efforts to develop and enhance recycling infrastructure | Hi | Med | (Blocktexx 2023) |
| 7 | Logan City Council Funding Programme – $1.5 Million | 2023 | Support industry infrastructure for recycling. | End-of-life | Financial Support | Blocktexx | Private Industry | City Council | Recycling Industry | Support efforts to develop and enhance recycling infrastructure | Hi | Med | (Blocktexx 2023) |

Table S.2 Summary of initiatives for textile waste management and the stage of life cycle addressed by European Union

The EU Green Deal is an ambitious framework that aims to make Europe carbon neutral by 2050. Within this overarching plan, the Circular Economy Action Plan (CEAP) is a key component, consisting of 35 targeted actions to drive the EU towards its climate goals. To achieve the objectives outlined in the CEAP for the textile sector, the EU Strategy for Sustainable and Circular Textiles was introduced. This strategy focuses on overhauling the entire lifecycle of textile products, from production to disposal, by setting regulations, frameworks, and mandatory compliance milestones. It aims to extend product lifespan, increase the use of recycled fibers, reduce waste, and promote sustainable consumption practices, ensuring that all sustainable textile frameworks align with these circular economy goals.

| No. | Name | Date | Description | Stage of Life Cycle Addressed | Type | Administrator / Beneficiary Organization | Entity Type | Scale | Typology | Target | Intensity-Targets | Intensity-Scope | Source |
| --- | --- | --- | --- | --- | --- | --- | --- | --- | --- | --- | --- | --- | --- |
| 1 | Ecodesign for Sustainable Products Regulation (Regulation (EU) 2024/1781) | 2024 | Establishes a framework for setting ecodesign requirements for sustainable products, improving product durability, repairability, recyclability, and addressing the presence of hazardous chemicals. | Design & Manufacturing, End-of-Life | Regulation | European Parliament, European Council / Manufacturers, Consumers, Retailers | Government / Multi-stakeholder Entity | European Union | Manufacturers, waste management | Improve product design for durability, repairability, recyclability, reducing waste, and promoting circularity through mandatory ecodesign requirements. | Hi | Hi | (Palma, Beltran et al. 2023) (Nickel 2024) |
| 1.1 | Digital Product Passport | 2024 | Electronically accessible digital identity for all products to make more informed decisions related to sustainability, circularity and regulatory compliance | Design & Manufacturing, use and end of life phase | Regulation | European Parliament, European Council / Manufacturers, Consumers, Retailers | Government / Multi-stakeholder Entity | European Union | Manufacturers, waste management | Allow custom authorities to perform automatic checks on the existence and authenticity of the DPPs of imported products | Hi | Hi | (Lehtisalo 2023) |
| 1.2 | Rules to address destruction of unsold consumer products | 2024 | Increase reuse and prevent resource destruction and maintain inventory for unsold products. | End-of-life | Regulation | European Parliament, European Council / Manufacturers, Consumers, Retailers | Government / Multi-stakeholder Entity | European Union | Retailers | To minimize resource waste that can potentially be reused and reduce raw material stress | Hi | Hi | (Rödig, Jepsen et al. 2021) |
| 1.3 | Green public procurement | 2024 | Mandating public authorities to purchase products that meet the highest levels of performance in terms of sustainability and circularity. | Design & Manufacturing and use | Regulation | European Parliament, European Council | Government / Multi-stakeholder Entity | European Union | Public Authorities | Ensure that public authorities spend for sustainable products | Hi | Hi | (Textile 2022) |
| 2 | Waste Framework Directive (WFD) | 2023 (amended)c | Sets basic concepts and definitions related to waste management, including definitions of waste, recycling, and recovery. It obliges Member States to separately collect textile waste by January 2025 and encourages the use of EPR schemes as an economic tool. | End-of-Life, Use Phase | Regulation | European Parliament, European Council / EU Member States | Government / Multi-stakeholder Entity | European Union | Textile Producers, Waste Management, Consumers | Ensure separate collection of textile waste by 2025 and encourage EPR schemes for better resource management. | Med | Hi | (Waste Framework Directive 2023) |
| 2.1 | Regulation on Shipments of Waste (EU 2024/1157) | 2024 | Regulates the export and import of waste, including textiles, to ensure environmentally sound management of waste, and prohibits the export of certain hazardous waste types to non-OECD countries. | End-of-Life | Regulation | European Parliament, European Council / Waste Management, Exporters | Government / Multi-stakeholder Entity | European Union | Waste Management, Exporters | Prohibit the export of hazardous waste and strengthen controls on non-hazardous waste exports, ensuring environmental protection in recipient countries. | Hi | Hi | (The European Parliament and of the Council), 2024 #169) |
| 3 | Unfair commercial practices directive (Directive (EU) 2024/825) | 2024 | A directive aimed at empowering consumers for the green transition by addressing unfair commercial practices, misleading claims, and ensuring product transparency in terms of durability, reparability, and recyclability. | Design & Manufacturing, Use Phase, End-of-Life | Regulation | European Parliament, European Council / Consumers, Manufacturers, Retailers | Government / Multi-stakeholder Entity | European Union | Consumers | Ensure better information for consumers on product durability, reparability, recyclability. Tackle greenwashing and misleading sustainability claims. | Hi | Hi | (The European Parliament and of the Council 2024) |
| 4 | Regulation on the registration, evaluation, authorization and restriction of chemicals (REACH) | 2021 (amended) | The REACH Regulatio is the EU's main framework for controlling the production, use, and disposal of chemicals. It aims to protect human health and the environment by ensuring that manufacturers and importers evaluate and manage the risks associated with chemical substances. It has a broad impact across industries, including textiles, by restricting chemicals that could affect the recyclability of materials | Raw material, manufacturing, and end-of-life | Regulations | European Chemicals Agency (ECHA) | Public-private partnership | European Union | Producers, recycler, importers | Under the REACH Regulation, any company that manufactures or imports chemical substances in quantities of 1 tonne or more per year must register these substances with the European Chemicals Agency (ECHA). This ensures that companies assess the risks associated with these chemicals and demonstrate how they manage them safely, thereby protecting human health and the environment. | Hi | Hi | (European Commission , Cha and Koo 2021) |
| 5 | Reset the Trend | 2023 | A campaign to raise awareness about sustainable fashion and fast fashion's environmental impact. The campaign engages young Europeans and promotes the EU Strategy for Sustainable and Circular Textiles. | Manufacturing and Use Phase | Awareness | European Commission | Public Sector | National | Consumers | Engage young Europeans in the battle against fast fashion and raise public awareness about the EU Strategy for Sustainable and Circular Textiles. | Low | Med | (Youth 4 Europe 2024) |

Table S.3 Summary of initiatives for textile waste management and the stage of life cycle addressed by France

| No | Name | Date | Description | Stage of Life Cycle Addressed | Type | Administrator / Beneficiary Organization | Entity Type | Scale | Typology | Target | Intensity-Scope | Intensity-Targets | Source |
| --- | --- | --- | --- | --- | --- | --- | --- | --- | --- | --- | --- | --- | --- |
| 1 | France’s Extended Producer Responsibility (EPR) Scheme for Textiles | 2007 | France was the first country to implement a legal EPR framework for managing textile waste, making producers responsible for their products’ end-of-life management. Refashion, a PRO, manages compliance with collection, recycling, and disposal. | End-of-Life | Regulation | Refashion / French Government | Public-Private Partnership | National | Textile Producers, Retailers, Waste Management | Reduce textile waste through recycling and reuse programs, funded by producers. Eco-modulation encourages use of sustainable materials. | Hi | Hi | (OECD Library 2016) |
| 2 | Anti-Waste and Circular Economy Law (Law no. 2020-105) | 2020 | A comprehensive French law aimed at eliminating waste, phasing out single-use plastics, reducing unsold goods destruction, encouraging reuse, promoting repairability, and extending the "Polluter Pays" principle. | End-of-Life, Manufacturing, Use Phase | Regualtion | French Government / Public Sector | Public Sector | National | All stakeholders across industries, including producers, retailers, and consumers | Phase out single-use plastics by 2040, introduce a repairability index, reduce waste, promote reuse, and ban destruction of unsold goods. | Hi | Hi | (Ellen Macarthur Foundation 2022) |
| 3 | French Climate & Resilience Law – Environmental Labelling for Products | 2023 | This law mandates environmental labelling for textiles and footwear, displaying environmental impacts such as greenhouse gas emissions and resource use across the product's lifecycle, ensuring transparency for consumers. | Manufacturing, Use Phase, End-of-Life | Regulation | French Government / ADEME | Public Sector | National | Producers, Retailers, Consumers | Make environmental labelling mandatory for fashion products, showing lifecycle impacts and aligning with EU standards. | Hi | Hi | (Lüttin 2024) |
| 4 | Repair Fund (Monnanni, Rimondi et al.) | 2022 | A repair fund that provides discounts (around 20%) on out-of-warranty repair costs, financed through Extended Producer Responsibility (EPR) fees paid by producers. | Use Phase | Financial Incentive | French Government / Refashion / EPR Fees | Public-Private Partnership | National | Consumers, Authorized Repairers | Reduce repair costs for consumers through a direct discount applied at the time of repair, promoting repair over disposal. | Hi | Hi | (Bajaj 2023) |

Table S.4 Summary of initiatives for textile waste management and the stage of life cycle addressed by Netherlands

| No | Name | Date | Description | Stage of Life Cycle Addressed | Type | Administrator / Beneficiary Organization | Entity Type | Scale | Typology | Target | Intensity-Scope | Intensity-Targets | Source |
| --- | --- | --- | --- | --- | --- | --- | --- | --- | --- | --- | --- | --- | --- |
| 1 | Extended Producer Responsibility (EPR) for Textiles | 2023 | Netherlands’ EPR for Textiles makes producers responsible for recycling and reusing textiles. It includes objectives for fiber-to-fiber recycling and reuse, and mandates reporting of textile volumes and recycling efforts. | End-of-Life | Regulation | Dutch Government / Rijkswaterstaat | Public Sector | National | Textile Producers, Importers | At least 50% of textiles must be recycled or reused by 2025, rising to 75% by 2030. Fiber-to-fiber recycling must account for 25% by 2025, increasing to 33% by 2030. | Hi | Hi | (Government of the Netherlands 2024) |
| 2 | National Circular Economy Programme (2023-2030) | 2023 | A national plan aimed at transitioning from a linear to a circular economy, with specific focus on the textile sector, aligned with the EU strategy and international goals such as the Paris Agreement. | Design, Manufacturing, End-of-Life | Roadmap | Dutch Government | Public Sector | National | Textile Producers, Retailers, Consumers | Circular economy goals include making 50% of textiles from sustainable/recycled materials by 2025 and achieving 100% recycling of non-reusable textiles by 2030. | Hi | Hi | (Government of the Netherlands 2020) |

Table S.5 Summary of initiatives for textile waste management and the stage of life cycle addressed by Latvia

| No | Name | Date | Description | Stage of Life Cycle Addressed | Type | Administrator / Beneficiary Organization | Entity Type | Scale | Typology | Target | Intensity-Scope | Intensity-Targets | Source |
| --- | --- | --- | --- | --- | --- | --- | --- | --- | --- | --- | --- | --- | --- |
| 1 | Latvia’s National Waste Prevention Programme 2021-2028 | 2021 | Latvia's national waste plan focuses on reducing waste generation, increasing recycling, and promoting repair, reuse, and eco-design. The plan integrates waste prevention actions for textiles. | End-of-Life, Use Phase | Regulation | Latvian Government / Ministry of Environmental Protection and Regional Development | Public Sector | National | Textile producers, consumers, repair shops | Reduce textile waste through reuse, repair, and circular strategies, with support for repair and reuse of textiles and footwear. | Med | Med | (Ministry of Smart Administration and Regional Development Republic of Latvia 2021) |
| 2 | Latvia’s Producer Responsibility Scheme for Textiles and Footwear (2023) | 2023 | Introduces mandatory producer responsibility for textiles and footwear, requiring businesses to collect, sort, and recycle textile waste. The scheme supports sustainable practices by promoting reuse and recycling of textiles. | End-of-Life | Regulation | Latvian Government / Ministry of Environmental Protection and Regional Development | Public-Private Partnership | National | Textile producers, retailers, waste management companies | Increase collection and recycling of textiles, promoting eco-friendly practices by manufacturers and ensuring recycling infrastructure development. | Hi | Hi | (Lizenzero 2024) |

Table S.6 Summary of initiatives for textile waste management and the stage of life cycle addressed by Germany

| No | Name | Date | Description | Stage of Life Cycle Addressed | Type | Administrator / Beneficiary Organization | Entity Type | Scale | Typology | Target | Intensity-Scope | Intensity-Targets | Source |
| --- | --- | --- | --- | --- | --- | --- | --- | --- | --- | --- | --- | --- | --- |
| 1 | Circular Economy Law | 2020 | Prohibited the destruction of unsold goods within Germany and mandated data transparency in the management of sold goods | End-of-life | Regulation | Government | Public-private | National | Textile Producers, Retailers, | The amendment will aid in transparency into the handling of unsold goods including textiles. | Med | Hi | (European Environmental Bureau 2023, Duiker 2024) |
| 2. | Separate collection of Textile waste | 2020 | Textile waste should be collected separately and must not be mixed with municipal solid waste. | End-of-life | Regulatory law | Government | Public | National | Consumers, council | The proposed law will require the separate collection of textile waste. The collected waste will then be sorted and sent for reuse or recycling | Hi | Hi | (Duiker 2024) |

S.7 Summary of initiatives for textile waste management and the stage of life cycle addressed by United Kingdom (UK)

| No | Name | Date | Description | Stage of Life Cycle Addressed | Type | Administrator / Beneficiary Organization | Entity Type | Scale | Typology | Target | Intensity-Scope | Intensity-Targets | Source |
| --- | --- | --- | --- | --- | --- | --- | --- | --- | --- | --- | --- | --- | --- |
| 1 | Textiles 2030: UK Sustainable Textile Action Plan | 2021 | A voluntary business program aimed at reducing the carbon and water footprint of the textiles industry by 50% and 30%, respectively, by 2030. Focus areas include design for circularity, circular business models, and closing the loop on materials. | Design, End-of-Life | Voluntary Circular Economy Program | UK Government / WRAP | Public-Private Partnership | National | Textile Producers, Retailers | Reduce carbon, water footprints, promote circularity | Hi | Med | (Department for Environment Food & Rural Affairs 2023) |
| 2 | UK Circular Fashion Programme | 2022 | A £15 million fund dedicated to driving circularity in the fashion and textiles sector, focusing on sorting, recycling, innovation, and research. The goal is to adopt scalable circular models by 2032. | End-of-Life | Circular Economy Innovation Program | UK Government / UK Research and Innovation | Public-Private Partnership | National | Textile Producers, Research Institutions, Universities | Create scalable circular business models and innovations in textile recycling | Hi | Med | (Department for Environment Food & Rural Affairs 2023) |
| 3 | Interdisciplinary Textiles Circularity Centre | 2022 | A £5.4 million research initiative to boost innovation in renewable textile materials, focusing on developing post-consumer textile products and using household waste as feedstock. | Design, End-of-Life | Circular Economy Research Program | UK Government / Academic Institutions | Public Sector | National | Research Institutions, Textile Producers | Drive research on renewable textile materials and circularity innovations | Med | Hi | (Department for Environment Food & Rural Affairs 2023) |

Table S.8 Summary of initiatives for textile waste management and the stage of life cycle addressed by Denmark

| No | Name | Date | Description | Stage of Life Cycle Addressed | Type | Administrator / Beneficiary Organization | Entity Type | Scale | Typology | Target | Intensity-Scope | Intensity-Targets | Source |
| --- | --- | --- | --- | --- | --- | --- | --- | --- | --- | --- | --- | --- | --- |
| 1 | Action Plan for Circular Economy (2021-2032) | 2023 | Textiles must be sorted separately from general waste across Denmark, ensuring they are reused or recycled instead of incinerated. Implementation is managed by local municipalities as part of the national circular economy strategy. | End-of-Life, Use Phase | Regulation | Danish Ministry of Environment / Municipalities | Public Sector | National | Textile producers, consumers, municipalities | Promote reuse and recycling of textile waste, aiming to reduce incineration and landfill. | Hi | Med | (Ministry of Environment of Denmark 2021) |

Table S.9 Summary of initiatives for textile waste management and the stage of life cycle addressed by Finland

| No | Name | Date | Description | Stage of Life Cycle Addressed | Type | Administrator / Beneficiary Organization | Entity Type | Scale | Typology | Target | Intensity-Scope | Intensity-Targets | Source |
| --- | --- | --- | --- | --- | --- | --- | --- | --- | --- | --- | --- | --- | --- |
| 1 | Finnish Law on Textile Waste Separate Collection (978/2021 Waste Decree) | 2023 | The Finnish law requires municipalities to arrange separate textile waste collection and prioritize reuse over recycling, starting from 2023. Finland is ahead of the EU mandate that requires this by 2025. | End-of-Life | Regulation | Finnish Government / Municipal Waste Authorities | Public Sector | National | Textile producers, municipalities, waste processors | Ensure the separate collection of textiles and promote reuse, with mechanical recycling of collected textiles where reuse is not possible. | Hi | Med | (Zero waste Europe 2023) |

Table S.10 Summary of initiatives for textile waste management and the stage of life cycle addressed by Luxembourg

| No | Name | Date | Description | Stage of Life Cycle Addressed | Type | Administrator / Beneficiary Organization | Entity Type | Scale | Typology | Target | Intensity-Scope | Intensity-Targets | Source |
| --- | --- | --- | --- | --- | --- | --- | --- | --- | --- | --- | --- | --- | --- |
| 1 | Luxembourg’s Textile Collection and Charity Reuse System | 2017 | Separate collection of textiles for charity by non-profits like Aide aux Enfants Handicapés du Grand Duché ASBL and Kolping Luxembourg ASBL. | End-of-Life | Regulation | Luxembourg Government / Non-Profits | Public-Private | National | Textiles, Shoes | Improve reuse of textiles and clothing | Hi | Med | (Ville De Luxembourg 2022) |

Table S.11 Summary of initiatives for textile waste management and the stage of life cycle addressed by Austria

| No | Name | Date | Description | Stage of Life Cycle Addressed | Type | Administrator / Beneficiary Organization | Entity Type | Scale | Typology | Target | Intensity-Scope | Intensity-Targets | Source |
| --- | --- | --- | --- | --- | --- | --- | --- | --- | --- | --- | --- | --- | --- |
| 1 | Action Plan for Circular Economy (2021-2032) | 2021 | Austria’s strategy aims to transition from a linear to circular economy, with specific goals for textiles including extending product life, promoting reuse, and increasing circular material use. | End-of-Life, Use Phase | Circular Economy Strategy | Austrian Government / Federal Ministry for Climate Action | Public Sector | National | National | Textile manufacturers, consumers, waste management companies, repair shops | Hi | Med | (Federal Ministry Republic of Austria 2020) |
| 2 | Repair Bonus Incentive (Federal Ministry Republic of Austria) | 2022 | A repair bonus that refunds citizens up to 50% of repair costs (up to €200), available for electrical appliances and other products. The scheme aims to reduce e-waste and promote repair over disposal. | Use Phase | Financial Incentive | Austrian Government / Funded by EU Recovery Fund | Public Sector | National | Consumer, Repair Shops | Subsidize repair costs by up to 50%, to encourage the repair of products rather than disposal, helping to reduce e-waste. | Hi | Hi | (Müncheberg 2024) |

Table S.12 Summary of initiatives for textile waste management and the stage of life cycle addressed by Indonesia

| No | Name | Date | Description | Stage of Life Cycle Addressed | Type | Administrator / Beneficiary Organization | Entity Type | Scale | Typology | Target | Intensity-Scope | Intensity-Targets | Source |
| --- | --- | --- | --- | --- | --- | --- | --- | --- | --- | --- | --- | --- | --- |
| 1 | Establishing Circular Textile Systems in Indonesia | 2024 | The roundtable focuses on creating a roadmap for a circular textile system in Indonesia, emphasizing recycling, reuse, and circular economy integration. | End-of-Life, Use Phase | Circular Economy Strategy | Global Fashion Agenda, Indonesian Government, NCFP, Brands | Public-Private | National | Textile producers, brands, recyclers | Promote recycling and reuse of textile waste through infrastructure development, stakeholder engagement, and investment. | Hi | Med | (Summit 2024) |
| 2 | Secondhand Clothing and Footwear Import Ban (2015) | 2015 | Indonesia banned secondhand clothing and footwear imports to protect its local textile industry from being undercut by cheap used clothing. This action supports local manufacturing by preventing competition from low-cost secondhand goods. | End-of-Life | Regulation | Indonesian Government / Ministry of Trade | Public Sector | National | Textile producers, retailers | Protect the local textile industry by preventing the import of used clothing and footwear, supporting local production and employment. | Hi | Hi | (BOF 2023) |

Table S.13 Summary of initiatives for textile waste management and the stage of life cycle addressed by South Korea

| No | Name | Date | Description | Stage of Life Cycle Addressed | Type | Administrator / Beneficiary Organization | Entity Type | Scale | Typology | Target | Intensity-Scope | Intensity-Targets | Source |
| --- | --- | --- | --- | --- | --- | --- | --- | --- | --- | --- | --- | --- | --- |
| 1 | South Korea’s Ban on Waste Textile Imports (2022) | 2022 | South Korea’s Ministry of Environment will ban the import of waste plastics, mixed waste papers, and waste textile fibers to prioritize domestic recycling and reduce dependency on foreign waste. This is part of a roadmap to reduce overall waste imports by 3.84 million tonnes by 2023. | End-of-Life | Regulation | South Korean Ministry of Environment | Public Sector | National | Textile producers, waste handlers | Ban imports of waste textile fibers to focus on utilizing domestic waste for recycling. | Hi | Hi | (Staub 2020) |

Table S.14 Summary of initiatives for textile waste management and the stage of life cycle addressed by China

| No | Name | Date | Description | Stage of Life Cycle Addressed | Type | Administrator / Beneficiary Organization | Entity Type | Scale | Typology | Target | Intensity-Scope | Intensity-Targets | Source |
| --- | --- | --- | --- | --- | --- | --- | --- | --- | --- | --- | --- | --- | --- |
| 1 | China Waste Import Ban | 2017 | China introduced a comprehensive ban on waste imports, including textiles, plastic, paper, and metals. The goal was to prioritize environmental protection and reduce dependency on foreign waste. The ban was implemented in phases, starting with specific materials in 2018 and expanding to all solid wastes by 2021. | End-of-Life | Import Ban | Chinese Ministry of Ecology and Environment (MEE) | Public Sector | National | All waste imports | Ban all textile waste and other solid wastes, promoting domestic recycling and environmental sustainability. | High: Nationwide scope covering all solid waste categories, including textiles. | High: Eliminates the import of textile waste, significantly reducing foreign waste inflow and enhancing domestic resource management. | (Hoogzaad 2019) |

Table S.15 Summary of initiatives for textile waste management and the stage of life cycle addressed by Turkey

| No | Name | Date | Description | Stage of Life Cycle Addressed | Type | Administrator / Beneficiary Organization | Entity Type | Scale | Typology | Target | Intensity-Scope | Intensity-Targets | Source |
| --- | --- | --- | --- | --- | --- | --- | --- | --- | --- | --- | --- | --- | --- |
| 1 | Turkey Government Policy on Recycled Material and Environmental Footprint (2030-2035) | 2021 | The government has launched a policy program aiming to use at least 30% recycled material in new clothing by 2030 and to halve the environmental footprint of the textile sector by 2035. | End-of-Life, Use Phase | Policy Directive | Turkish Government / Textile Industry | Public-Private | National | Textile producers, recyclers, industry | Promote the use of recycled materials and significantly reduce the environmental impact of textile production. | Hi | Med | (Ministry of Foreign Affairs 2021) |

Table S.16 Summary of initiatives for textile waste management and the stage of life cycle addressed by Saudi Arabia

| No | Name | Date | Description | Stage of Life Cycle Addressed | Type | Administrator / Beneficiary Organization | Entity Type | Scale | Typology | Target | Intensity-Scope | Intensity-Targets | Source |
| --- | --- | --- | --- | --- | --- | --- | --- | --- | --- | --- | --- | --- | --- |
| 1 | Ihram Recycling Project | 2023 | A circular economy initiative to recycle Ihrams, the cotton garments worn by male pilgrims during Hajj and Umrah. In 2023, 34 tons of Ihrams were collected for recycling, with plans to scale up collection to 227 tons in future seasons. | End-of-Life, Use Phase | Circular Economy Initiative | Saudi Fashion Commission, Saudi Investment Recycling Company (SIRC), Royal Commission of Makkah City, ISKO, Tadweem | Public-Private | National | Textile producers, recyclers, pilgrims | Promote recycling of Ihrams as part of broader textile circularity efforts, with a goal of reducing textile waste and carbon emissions. | Hi | Med | (Fashion Comission 2024) |

Table S.17 Summary of governmental initiatives for textile waste management and the stage of life cycle addressed in North America

| No | Name | Date | Description | Stage of Life Cycle Addressed | Type | Administrator / Beneficiary Organization | Entity Type | Scale | Typology | Target | Intensity-Scope | Intensity-Targets | Source |
| --- | --- | --- | --- | --- | --- | --- | --- | --- | --- | --- | --- | --- | --- |
| 1 | California Responsible Textile Recovery Act (SB 707) | 2024 | Signed into law on September 22, 2024, this Act requires manufacturers and distributors to participate in an extended producer responsibility (EPR) program for apparel and textile products, including clothing, footwear, linens, and accessories. | End-of-Life, Use Phase | Extended Producer Responsibility (EPR) | California Government / CalRecycle | Public-Private | State-Wide | Textile producers, retailers, recyclers | Mandates the creation of a statewide collection, recycling, and repair program for textiles, including PFAS management in the recycling process. | Hi | Med | (Danielle N. Garno 2024) |
| 2 | California SB 1187 Fabric Recycling Pilot Project | 2024 | This bill establishes a pilot project in Los Angeles and Ventura Counties to study and report on the feasibility of recycling fabric. The project will 3partner with garment manufacturers and focus on creating a circular economy for textiles. The project will report annually on the amount of textiles collected and diverted from disposal. | End-of-Life, Use Phase | Pilot Recycling Project | California Department of Resources Recycling and Recovery | Public-Private | Local | Garment manufacturers, recyclers | Study the feasibility of recycling fabrics and diverting textile waste, contributing to a circular economy for textiles. | Med | Med | (Digital Democracy Calmatters 2021) |
| 3 | New York City Textiles Separation Requirement | 2023 | Businesses are required to separate and recycle or repurpose textile waste if textiles make up more than 10% of their waste. Includes items such as clothing, fabric scraps, shoes, and curtains. | End-of-Life | Recycling Law | New York City Department of Sanitation, donateNYC, refashionNYC | Public Sector | City-Wide | Textile producers, recyclers, businesses | Promote textile recycling and reuse through mandatory separation, supported by donateNYC and refashionNYC programs. | Hi | Med | (New York City Department of Sanitation 2020) |
| 4 | Massachusetts Clothing and Textile Recovery Initiative | 2022 | Massachusetts banned the disposal of textiles, including clothing, footwear, bedding, curtains, and fabric, starting November 2022. Textiles must be reused, repurposed, or recycled if they are clean and dry, with support for donation options. | End-of-Life | Waste Disposal Ban | Massachusetts Department of Environmental Protection (MassDEP) | Public Sector | State-Wide | Textile producers, recyclers, residents | Reduce textile waste and promote reuse and recycling through the ban and recovery programs like "Beyond the Bin". | Hi | Med | (Massachusetts Department of Environmental Protection 2022) |

Table S.18 Summary of governmental initiatives for textile waste management and the stage of life cycle addressed in Africa

| No | Name | Date | Description | Stage of Life Cycle Addressed | Type | Administrator / Beneficiary Organization | Entity Type | Scale | Typology | Target | Intensity-Scope | Intensity-Targets | Source |
| --- | --- | --- | --- | --- | --- | --- | --- | --- | --- | --- | --- | --- | --- |
| 1 | African Union Climate Change and Resilient Development Strategy Action Plan | 2022–2032 | Envisions a climate-resilient Africa, focusing on strengthening waste value chains, promoting waste-to-resource conversion, and circular economy systems. Includes national industrial waste management programs. | Waste Management | Policy Framework | African Union | Government | Continental | Circular Economy | Climate resilience, emission reduction | Hi | Hi | (Bukhari, Carrasco-Gallego et al. 2018) |
| 2 | African Union Continental Circular Economy Action Plan (CEAP) | 2024–2034 | Prioritizes sectors such as textiles, construction, packaging, plastics, and mining, aiming to foster a circular economy across the continent. | Multiple Stages (Production to Waste) | Action Plan | African Union | Government | Continental | Circular Economy | Promotion of circular economy principles in prioritized sectors | Expands circular economy priorities to textiles and other sectors. | Sector-specific prioritization for circularity. | (Oxford Economics 2024) |
| 3 | Ghana Circular Economy Action Plan (CEAP) (Under Development) | TBD | Prioritizes textiles, plastics, electronics, agriculture, built environment, and water systems; focuses on developing circular economy principles. | Multiple Stages | Action Plan | Republic of Ghana | Government | National | Circular Economy | Enhancing circular economy in prioritized sectors | Under development; potential focus on sector-specific actions, including textiles. | Not yet defined. | (Quashie-Idun 2024) |
| 4 | Ghana National Solid Waste Management Strategy | 2020 | Addresses urban waste management crises with a focus on public behavior and service delivery; emphasizes opportunities for recycling and circular economy development. | Waste Management | Strategy | Republic of Ghana | Government | National | Waste Management | Reducing urban waste and fostering recycling opportunities | Highlights need for infrastructure development; links waste management to recycling opportunities. | No specific targets for textiles but broader focus on urban waste systems. | (Bukhari, Carrasco-Gallego et al. 2018) |
| 5 | Kenya Sustainable Waste Management Act | 2022 | Outlines responsibilities of private and public sectors to ensure a clean environment; promotes infrastructure development for recycling and local circular economy. | Waste Management | Legislation | Government of Kenya | Government | National | Circular Economy / Waste Policy | Realizing citizens' right to a clean and healthy environment. | Establishes framework for private-public sector roles in waste management. | Targets infrastructure development and capacity building. | (Bukhari, Carrasco-Gallego et al. 2018) |
| 6 | Kenya Green Economy Strategy and Implementation Plan | 2016–2030 | Focuses on capacity development to prevent waste generation and enhance recycling. | Waste Management | Strategy | Government of Kenya | Government | National | Green Economy | Capacity development and waste prevention. | Promotes integration of green economy principles, including textiles. | Capacity-focused, no specific numerical targets. | (Bukhari, Carrasco-Gallego et al. 2018) |
| 7 | Kenya Sustainable Waste Management Policy | 2021 | Creates enabling environment for waste management; focuses on employment opportunities and local circular economy development. | Waste Management | Policy Framework | Government of Kenya | Government | National | Circular Economy | Reducing waste generation and improving employment opportunities. | Encourages private sector participation; integrates circular economy principles. | No explicit targets for textile-specific actions. | (Bukhari, Carrasco-Gallego et al. 2018) |
| 8 | Mozambique Green Economy Action Plan (GEAP) | 2013 | Implements roadmap objectives with concrete actions for integrating green economy principles into national development priorities. | Multiple Stages | Action Plan | Government of Mozambique | Government | National | Green Economy | National green economy agenda integration. | Focus on integrating green economy into national planning processes. | Broader environmental goals, no textile-specific indicators. | (Bukhari, Carrasco-Gallego et al. 2018) |
| 9 | Mozambique Green Economy Roadmap | 2012 | Lays foundation for green economy development; includes integrating green economy goals into public planning and budgeting. | Multiple Stages | Roadmap | Government of Mozambique | Government | National | Green Economy | Promoting green growth through public policy integration. | Provides framework for aligning green economy goals with national planning. | General green economy goals, no textile-specific targets. | (Bukhari, Carrasco-Gallego et al. 2018) |
| 10 | Uganda Ban on Textile Waste Import and Secondhand Clothing | 2023 | In September 2023, Uganda implemented a ban on the import of textile waste and secondhand clothing to protect the local textile industry and encourage domestic production and recycling efforts. | End-of-Life | Import Ban | Uganda Government / Ministry of Trade and Industry | Public Sector | National | Textile producers, retailers | Reduce reliance on secondhand imports, promote local textile production and recycling. | Hi | low | (Kolade 2023) |
| 11 | Rwanda Ban on Secondhand Clothing | 2018 | In 2018, Rwanda became the first country in East Africa to impose a ban on the import of secondhand clothes, focusing on rebuilding its textile industry and supporting local textile production. | End-of-Life | Import Ban | Rwanda Government / Ministry of Trade and Industry | Public Sector | National | Textile producers, retailers | Revive the domestic textile industry, reduce reliance on secondhand clothing imports. | Hi | Med | (Africanews 2019) |

Africanews. (2019). "Rwanda, Tanzania and Uganda face US sanction after used clothes ban." from <https://www.africanews.com/2017/06/21/rwanda-tanzania-and-uganda-face-us-sanction-after-used-clothes-ban//>.

Australian Government. (2021). "Entrepreneurs' Programme - Accelerating Commercialisation." from <https://www.grants.gov.au/Ga/Show/0e59d6ab-6d51-421b-a355-becda02aefef>.

Bajaj, N. (2023). "France Will Pay for Your Clothing Repairs." from <https://www.thecommons.earth/blog/france-will-pay-for-your-clothing-repairs#:~:text=Starting%20in%20November%202023%2C%20residents,resoling%20shoes%20or%20mending%20clothes.&text=This%20measure%20will%20make%20it,shoes%20instead%20of%20discarding%20them>.

Blocktexx. (2023). "Textile recycling business to divert 50,000 tonnes of landfill waste and create 140 jobs over four years." from <https://www.blocktexx.com/news/textile-recycling-business-to-divert-50000-tonnes-of-landfill-waste-and-create-140-jobs-over-four-years>.

BOF. (2023). "Indonesia Cracks Down on Used Clothing Imports." from <https://www.businessoffashion.com/news/global-markets/indonesia-cracks-down-on-used-clothing-imports/#:~:text=Indonesia%20prohibited%20secondhand%20clothing%20and,made%20the%20ban%20largely%20ineffective>.

Bukhari, M. A., R. Carrasco-Gallego and E. Ponce-Cueto (2018). "Developing a national programme for textiles and clothing recovery." Waste Management & Research **36**(4): 321-331.

CEAP. (2024). "Circular economy action plan." from <https://environment.ec.europa.eu/strategy/circular-economy-action-plan_en>.

Cha, Y. and M. G. Koo (2021). "Who embraces technical barriers to trade? The case of European REACH regulations." World Trade Review **20**(1): 25-39.

Danielle N. Garno, V. Y. (2024). "A Closer Look at California's Recently Passed Responsible Textile Recovery Act of 2024." from <https://www.hklaw.com/en/insights/publications/2024/10/a-closer-look-at-californias-recently-passed-responsible#:~:text=With%20Gov.%20Gavin%20Newsom's%20signature,U.S.%20EPR%20is%20an%20environmental>.

Department for Environment Food & Rural Affairs. (2023). "The waste prevention programme for England: Maximising Resources, Minimising Waste." from <https://www.gov.uk/government/publications/waste-prevention-programme-for-england-maximising-resources-minimising-waste/the-waste-prevention-programme-for-england-maximising-resources-minimising-waste#:~:text=Our%20aim%3A%20to%20support%20our,a%20profitable%20textile%20recycling%20industry>.

Digital Democracy Calmatters. (2021). "SB 1187: Fabric recycling: pilot project." from <https://digitaldemocracy.calmatters.org/bills/ca_202120220sb1187>.

Duiker, I. (2024). "Tackling Textile Waste in Europe – Germany, France and Italy under the loop." from <https://dress-ecode.com/en/2024/09/23/tackling-textile-waste-in-europe-germany-france-and-italy-under-the-loop/>.

Ellen Macarthur Foundation. (2022). "France’s Anti-waste and Circular Economy Law." from <https://www.ellenmacarthurfoundation.org/circular-examples/frances-anti-waste-and-circular-economy-law>.

ESP Policy. (2024). "Environmentally Sustainable Procurement Policy and Reporting Framework." from <https://www.dcceew.gov.au/environment/protection/waste/sustainable-procurement/environmentally-sustainable-procurement-policy>.

European Commission. (2007). "REACH Regulation." from <https://environment.ec.europa.eu/topics/chemicals/reach-regulation_en>.

European Environmental Bureau. (2023). "Policy brief on prohibiting the destruction of unsold goods." from <https://eeb.org/wp-content/uploads/2021/10/Prohibiting-the-destruction-of-unsold-goods-Policy-brief-2021.pdf>.

Fashion Comission. (2024). "Ihram Circularity: Weaving a Greener Hajj and Umrah Experience." from <https://saudi-fashion-co.objects.frb.io/Ihram-Circularity-Weaving-a-Greener-Hajj-and-Umrah-Experience-English.pdf>.

Federal Ministry Republic of Austria. (2020). "The Austrian Circular Economy Strategy Austria on the path to a sustainable and circular society." from <https://www.bmk.gv.at/en/topics/climate-environment/waste-resource-management/ces.html>.

Government of the Netherlands. (2020). "Policy programme for circular textile 2020-2025." from <https://www.government.nl/documents/parliamentary-documents/2020/04/14/policy-programme-for-circular-textile-2020-2025>.

Government of the Netherlands. (2024). "Infographic: extended producer responsibility for textiles." from <https://www.government.nl/documents/publications/2023/05/01/infographic-extended-producer-responsibility-for-textiles>.

Hoogzaad, J. (2019). "Chinese waste ban forcing a breakthrough in circular economy ambitions in Australia." from <https://www.shiftingparadigms.nl/projects/chinese-waste-ban-forcing-a-breakthrough-in-circular-economy-ambitions-in-australia/#:~:text=Announced%20in%202017%2C%20China%20de,expanded%20to%2032%20waste%20types>.

Kolade, B. (2023). "A ban on used clothing imports isn’t the answer – Uganda must find homegrown solutions." from <https://www.theguardian.com/global-development/2023/sep/07/a-ban-on-used-clothing-imports-isnt-the-answer-uganda-must-find-homegrown-solutions>.

Landrigan, P. J., H. Raps, M. Cropper, C. Bald, M. Brunner, E. M. Canonizado, D. Charles, T. C. Chiles, M. J. Donohue, J. Enck, P. Fenichel, L. E. Fleming, C. Ferrier-Pages, R. Fordham, A. Gozt, C. Griffin, M. E. Hahn, B. Haryanto, R. Hixson, H. Ianelli, B. D. James, P. Kumar, A. Laborde, K. L. Law, K. Martin, J. Mu, Y. Mulders, A. Mustapha, J. Niu, S. Pahl, Y. Park, M. L. Pedrotti, J. A. Pitt, M. Ruchirawat, B. J. Seewoo, M. Spring, J. J. Stegeman, W. Suk, C. Symeonides, H. Takada, R. C. Thompson, A. Vicini, Z. Wang, E. Whitman, D. Wirth, M. Wolff, A. K. Yousuf and S. Dunlop (2023). "The Minderoo-Monaco Commission on Plastics and Human Health." Ann Glob Health **89**(1): 23.

Lehtisalo, E. (2023). "Digital Product Passport for Textile and Fashion Enterprises: Opportunities and Challenges."

Lizenzero. (2024). "Textile EPR in Europe: an opportunity for a greener future in fashion." from <https://www.lizenzero.eu/en/blog/textile-epr-in-europe-an-opportunity-for-a-greener-future-in-fashion/#:~:text=Introduction%20of%20the%20textile%20EPR%20in%20Latvia&text=The%20amendment%20to%20the%20law,fees%20to%20an%20EPR%20system>.

Lüttin, L. (2024). "France’s Climate and Resilience Law for Fashion & Textile Companies." from <https://www.carbonfact.com/blog/policy/france-climate-and-resilience-law#:~:text=Labeling%20requirements%3A%20At%20the%20time,impact%20on%20biodiversity%2C%20and%20the>.

Massachusetts Department of Environmental Protection. (2022). "Clothing and Textile Recovery." from <https://www.mass.gov/guides/clothing-and-textile-recovery#:~:text=In%20November%202022%2C%20Massachusetts%20banned,donated%20to%20a%20textile%20recycler>.

Ministry of Environment of Denmark. (2021). "Action plan for Circular Economy ", from <https://ddrn.dk/wp-content/uploads/2024/01/alle-faktaark-1.pdf>.

Ministry of Foreign Affairs. (2021). "DEFINING CIRCULARITY OF TEXTILE INDUSTRY IN TURKEY." from <https://www.rvo.nl/sites/default/files/2021/04/Circulair%20Textiles%20Turkey%202021.pdf>.

Ministry of Smart Administration and Regional Development Republic of Latvia. (2021). "Minister Plešs: State waste management plan will ensure the development of the sector." from <https://www.varam.gov.lv/en/article/minister-pless-state-waste-management-plan-will-ensure-development-sector?utm_source=https%3A%2F%2Fwww.google.com%2F>.

Monnanni, A., V. Rimondi, G. Morelli, A. Nannoni, A. Cincinelli, T. Martellini, D. Chelazzi, M. Laurati, L. Sforzi, F. Ciani, P. Lattanzi and P. Costagliola (2024). "Microplastics and microfibers contamination in the Arno River (Central Italy): Impact from urban areas and contribution to the Mediterranean Sea." Science of The Total Environment **955**: 177113.

Müncheberg, M. (2024). "New EU rules for sustainable consumer behavior, the repair bonus is already available now." from <https://www.sea-help.eu/en/news-general/eu-repair-bonus-replacement/#:~:text=In%20Austria%20is%20already%20further,of%20200%20euros)%20for%20their>.

New York City Department of Sanitation. (2020). "Textiles Separation." from <https://www.nyc.gov/site/dsny/businesses/materials-handling/textiles.page>.

Nickel, L. (2024). "Ecodesign for Sustainable Products Regulation (ESPR)." from <https://ecochain.com/blog/espr-2024-overview/>.

OECD Library. (2016). "20 years of EPR in France: Achievements, lessons learned and challenges ahead." from <https://www.oecd-ilibrary.org/docserver/9789264256385-15-en.pdf?expires=1729419668&id=id&accname=guest&checksum=76AC8EE4559B64EE4B3167EE05623D39>.

Oxford Economics. (2024). "The socioeconomic impact of second-hand clothes in Africa and the EU27+." from <https://www.oxfordeconomics.com/resource/the-socioeconomic-impact-of-second-hand-clothes-in-africa-and-the-eu27/>.

Palma, R., B. Beltran, L. Brotons, P. Arriba and S. Mengual (2023). "Ecodesign fo r Sustainable Products Regulation-Preliminary Study on New Product Prioritie s Technical Report (Draft) Circular Economy and Sustainable Industry."

Quashie-Idun, S. (2024). "Return to sender: Why Africa doesn’t need any more of your clothes." from <https://www.greenpeace.org/africa/en/blog/54827/return-to-sender-why-africa-doesnt-need-any-more-of-your-clothes/>.

Queensland Government. (2024). "Project Boomerang - Salvos Stores textile recycling hub." from <https://www.qld.gov.au/environment/circular-economy-waste-reduction/funding-grants/salvos-textile-recycling>.

Rödig, L., D. Jepsen, T. Zimmermann, R. Memelink and A. Falkenstein. (2021). "Policy Brief on Prohibiting the destruction of unsold goods." from <https://eeb.org/library/prohibiting-the-destruction-of-unsold-goods/>.

Seamless Report. (2023). "Scheme design summary report." from <https://www.seamlessaustralia.com/news/seamless-design-summary-report>.

Staub, C. (2020). "South Korea to enact import restrictions." from <https://resource-recycling.com/recycling/2020/03/10/south-korea-to-enact-import-restrictions/>.

Summit, G. F. (2024). "Recap of Round Table on: Establishing Circular Textile Systems in Indonesia." from <https://globalfashionagenda.org/wp-content/uploads/2024/07/240604-Meeting-Report-Roundtable-Establishing-Circular-Textile-Systems-in-Idonesia.pdf>.

Textile. (2022). " EU Strategy for Sustainable and Circular Textiles." from <https://environment.ec.europa.eu/publications/textiles-strategy_en>.

The European Parliament and of the Council (2024). Directive (EU) 2024/825 of the European Parliament and of the Council of 28 February 2024 amending Directives 2005/29/EC and 2011/83/EU as regards empowering consumers for the green transition through better protection against unfair practices and through better information.

The European Parliament and of the Council (2024). REGULATION (EU) 2024/1157 OF THE EUROPEAN PARLIAMENT AND OF THE COUNCIL of 11 April 2024 on shipments of waste, amending Regulations (EU) No 1257/2013 and (EU) 2020/1056 and repealing Regulation (EC) No 1013/2006.

Victoria, M. i. (2024). "Manufacturing support." from <https://djsir.vic.gov.au/made-in-victoria/manufacturing-support#:~:text=%244%20million%20investment%20to%20ensure,materials%20to%20ensure%20workplace%20safety>.

Ville De Luxembourg. (2022). "Clothing and Textile." 2022, from <https://www.vdl.lu/en/living/your-home/waste-collection-and-sorting/type-waste/clothing-and-textiles>.

Waste Framework Directive. (2023). "Implementation of the Waste Framework Directive." from <https://environment.ec.europa.eu/topics/waste-and-recycling/implementation-waste-framework-directive_en>.

Youth 4 Europe. (2024). "ReSet the Trend, Embrace Sustainable Fashion." from <https://youthforeurope.eu/reset-the-trend-embrace-sustainable-fashion/>.

Zero waste Europe. (2023). "The Finnish law on textile waste separate collection and implementation pilots." from <https://zerowasteeurope.eu/library/the-finnish-law-on-textile-waste-separate-collection-and-implementation-pilots/>.
